# Phage resistance formation and fitness costs of hypervirulent *Klebsiella pneumoniae* mediated by K2 capsule-specific phage and the corresponding mechanisms

**DOI:** 10.3389/fmicb.2023.1156292

**Published:** 2023-07-19

**Authors:** Miran Tang, Zeyu Huang, Xiaodong Zhang, Jingchun Kong, Beibei Zhou, Yijia Han, Yi Zhang, Lijiang Chen, Tieli Zhou

**Affiliations:** ^1^Department of Clinical Laboratory, The First Affiliated Hospital of Wenzhou Medical University and Key Laboratory of Clinical Laboratory Diagnosis and Translational Research of Zhejiang Province, The First Affiliated Hospital of Wenzhou Medical University, Wenzhou, Zhejiang, China; ^2^Department of Medical Lab Science, School of Laboratory Medicine and Life Science, Wenzhou Medical University, Wenzhou, Zhejiang, China

**Keywords:** bacteriophage, phage resistance, hypervirulent *Klebsiella pneumoniae*, capsular polysaccharides, lipopolysaccharides, polysaccharide depolymerase, fitness cost, virulence

## Abstract

**Introduction:**

Phage is promising for the treatment of hypervirulent *Klebsiella pneumoniae* (hvKP) infections. Although phage resistance seems inevitable, we found that there still was optimization space in phage therapy for hvKP infection.

**Methods:**

The clinical isolate K. pneumoniae FK1979 was used to recover the lysis phage ΦFK1979 from hospital sewage. Phage-resistant bacteria were obtained on LB agar and used to isolate phages from sewage. The plaque assay, transmission electron microscopy (TEM), multiplicity of infection test, one-step growth curve assay, and genome analysis were performed to characterize the phages. Colony morphology, precipitation test and scanning electron microscope were used to characterize the bacteria. The absorption test, spot test and efficiency of plating (EOP) assay were used to identify the sensitivity of bacteria to phages. Whole genome sequencing (WGS) was used to identify gene mutations of phage-resistant bacteria. The gene expression levels were detected by RT-qPCR. Genes knockout and complementation of the mutant genes were performed. The change of capsules was detected by capsule quantification and TEM. The growth kinetics, serum resistance, biofilm formation, adhesion and invasion to A549 and RAW 264.7 cells, as well as G. mellonella and mice infection models, were used to evaluate the fitness and virulence of bacteria.

**Results and discussion:**

Here, we demonstrated that K2 capsule type sequence type 86 hvKP FK1979, one of the main pandemic lineages of hvKP with thick capsule, rapidly developed resistance to a K2-specific lysis phage ΦFK1979 which was well-studied in this work to possess polysaccharide depolymerase. The phage-resistant mutants showed a marked decrease in capsule expression. WGS revealed single nucleotide polymorphism (SNP) in genes encoding RfaH, galU, sugar glycosyltransferase, and polysaccharide deacetylase family protein in the mutants. RfaH and galU were further identified as being required for capsule production and phage sensitivity. Expressions of genes involved in the biosynthesis or regulation of capsule and/or lipopolysaccharide significantly decreased in the mutants. Despite the rapid and frequent development of phage resistance being a disadvantage, the attenuation of virulence and fitness *in vitro* and *in vivo* indicated that phage-resistant mutants of hvKP were more susceptible to the immunity system. Interestingly, the newly isolated phages targeting mutants changed significantly in their plaque and virus particle morphology. Their genomes were much larger than and significantly different from that of ΦFK1979. They possessed much more functional proteins and strikingly broader host spectrums than ΦFK1979. Our study suggests that K2-specific phage has the potential to function as an antivirulence agent, or a part of phage cocktails combined with phages targeting phage-resistant bacteria, against hvKP-relevant infections.

## 1. Introduction

*Klebsiella pneumoniae* (*K. pneumoniae*) is an important clinically relevant opportunistic pathogen that causes a wide variety of infections. Over the past three decades, hypervirulent *K. pneumoniae* (hvKP) has emerged as a major cause of life-threatening invasive community-acquired infections in the healthy population (Liu et al., 2014). HvKPs are usually hypermucoviscous attributed to their overproduction of capsular polysaccharides (CPS; K-antigen; Feldman et al., 2019). Among at least 134 capsular types, serotypes K1 and K2 have been identified as the most predominant and virulent capsular types with epidemiological importance (Follador et al., 2016). Being much more invasive than classic *K. pneumoniae*, as well as with the risk of the convergence of hypervirulence and multidrug resistance causing untreatable invasive infections conferred by traditional antimicrobials selection pressure (Liu et al., 2014), HvKPs, especially the popular serotypes K1 and K2, are top priority pathogens requiring urgent development of new therapeutic strategies.

Bacteriophages (“phages,” viruses that infect bacteria) carry the potential to control hvKP infections as a promising alternative for antibiotics. Due to its bacteriolytic activity and bacterial host specificity, phage therapy can simultaneously overcome two serious side effects: drug resistance emergence and symbiont disorders (Bhargava et al., 2021). More importantly, hvKP is characterized by high production of extracellular polysaccharides, including exopolysaccharides, CPS, and lipopolysaccharides (LPS), which have been described as the absorption receptors for phages, especially those that possess specific polysaccharide depolymerase (Rice et al., 2021; Chen et al., 2022).

However, phage resistance has been illustrated as a threat to undermine phage therapy and seems inevitable (Hesse et al., 2020). Recently, more and more studies have illustrated the contribution of the genetic diversity and genomic rearrangements of bacterial surface receptors to this (Guinane et al., 2011; Li et al., 2018; Tan D. et al., 2020; Kaszowska et al., 2021; Egido et al., 2022). However, the specific resistance mechanisms of hvKP mediated by K2 capsule-specific phage have not been clarified. In addition, fitness costs triggered by phage resistance in hvKP are not fully studied. Clarifying these questions will aid in turning phage resistance into a therapeutic advantage and engineering sustainable phage-based antivirulence approaches or phage cocktails to fight hvKP infections.

In this study, we isolated and characterized a lysis phage ΦFK1979 able to specifically lyse K2 hvKP, expanding the repertoire of phages against hvKP. It was revealed by morphological, biological, and genomic characteristics analysis that the host *K. pneumoniae* FK1979 is a typical K2 ST86 hvKP strain with high production of CPS, and ΦFK1979 possesses K2-specific polysaccharide depolymerase. We characterized phage-resistant mutants of hvKP FK1979 mediated by ΦFK1979 and their phage counterparts, as well as the associated fitness costs. Emphasis is placed on the corresponding phage resistance mechanisms. This study suggests that K2-specific phage has the potential to function as an antivirulence agent, or a part of phage cocktails combined with other phages with distinct receptors, against hvKP-relevant infections.

## 2. Materials and methods

### 2.1. Isolation and purification of phages

Phages were isolated from raw sewage samples from a hospital in eastern China. Briefly, aliquots of sewage were combined with overnight cultures of FK1979 and incubated in 5 × LB medium for 24 h at 37°C to enrich phages. Lysates were purified until the phage plaque morphology is consistent. The procedures were the same for the isolation and purification of phages targeting mutants resistant to ΦFK1979.

### 2.2. Transmission electron microscope

For the morphology of phage observation, a 10-μL prepared phage suspension sample was dropped on the copper grids with carbon-coated ultrathin formvar film (SolelyBio., China). Negative staining was performed with a phosphotungstate dye, and the grids were placed under the Transmission electron microscope (TEM; Hitachi HT7800) to examine the morphology of the phages. For bacterial capsule polysaccharide visualization of gene deletion and complement mutants, bacterial precipitation was fixed with 2.5% (v/v) glutaraldehyde (Aladdin, China) at 4°C overnight, and then treated with 1% OsO4 in 0.1 M PBS for 1 h. Subsequently, samples were washed with 0.1 M PBS and then dehydrated using acetone. Finally, samples were flat-embedded and 70-nm sections were obtained by longitudinal or vertical sectioning of the sample. Sections were post-stained with uranyl acetate and lead citrate. Micrographs were taken by TEM (JEM1200EX/JEM1010).

### 2.3. Scanning electron microscope

Scanning electron microscope (SEM) was performed for capsule polysaccharide visualization of phage-resistant mutants. A 20 μL droplet of sterile phosphate-buffered saline (PBS)-washed bacterial suspension was placed onto a fresh gold-coated silicon wafer and then fixed with 2.5% (v/v) glutaraldehyde (Aladdin, China) at 4°C overnight. After drying, the samples were dehydrated by 20, 40, 70, 90, 95, and 100% (v/v) of ethanol. Residual ethanol was removed with a critical-point dryer CPD300. Finally, the samples were gold sputtered and observed by SEM (Hitachi SU8010).

### 2.4. Optimal multiplicity of infection of phage

The optimal multiplicity of infection (OMOI) of phage was determined as previously described (Pertics et al., 2021). The fresh culture of bacteria was mixed with equal volumes of diluted phage (1 × 10^3^–1 × 10^10^) plaque-forming units (PFUs)/mL at the MOI as 0.00001, 0.0001, 0.001, 0.01, 0.1, 1, 10, and 100. Mixtures were cultured at 37°C for 5 h, then phage titer was determined using the double-layer plate method.

### 2.5. One-step growth curve assay

A one-step growth curve assay was performed as previously described (Pertics et al., 2021). The phage was mixed with exponential phase FK1979 (0.5 McF diluted 10 folds, i.e., 1.5 × 10^7^ CFUs/mL) at the MOI of 0.0001, and was incubated at 37°C for 10 min to allow maximum adsorption of phage. Subsequently, the mixture was centrifuged at 10,000 g (4°C) for 10 min to remove unabsorbed phage. Then, the supernatant was discarded, and the pellets were washed and suspended with LB broth. These samples were incubated at 37°C and taken at indicated time points. The phage titers were measured using the double-layer agar method.

### 2.6. Phage DNA extraction, whole genome sequencing, and bioinformatics analysis

Genomic DNA was extracted using a phage genome extraction kit (ABigen, China). Whole genome sequencing (WGS) was performed at the Shanghai Personal Biotechnology Co., Ltd. on an Illumina NovaSeq PE250 platform (Illumina, United States). ORFs were predicted by GeneMarkS program (Besemer et al., 2001). Genome annotation was performed by the RAST server (https://rast.nmpdr.org/, 25 February 2022), the PHASTER online website (https://phaster.ca/submissions/ZZ_edeacd9cd3#!, 2 March 2022) and the prokka online website (https://proksee.ca/, 3 March 2022). Virulence and antibiotic-resistant genes of phage were predicted by VFDB (http://www.mgc.ac.cn/VFs/search_VFs.htm) and CGE resFinder model (https://cge.food.dtu.dk/services/ResFinder/). Prediction of the phage life cycle was computationally analyzed using the PHACTS software (McNair et al., 2012; Benler et al., 2018). Snapgene v4.1.8 was used to illustrate the genome map. Homology searches were conducted by the BLAST tools at the NCBI website (https://blast.ncbi.nlm.nih.gov/Blast.cgi, 18 November 2021). The whole genome phylogenetic tree was created using the Genome-BLAST Distance Phylogeny method in VICTOR (https://ggdc.dsmz.de/victor.php, 8 May 2022). Phage was classified according to the guidelines on the ICTV (https://talk.ictvonline.org/taxonomy/, 19 May 2022). Protein homology was conducted by NCBI BLASTp (https://blast.ncbi.nlm.nih.gov/Blast.cgi?PROGRAM=blastp&PAGE_TYPE=BlastSearch&LINK_LOC=blasthome, 19 May 2022), and Identify Conserved Domains online website of NCBI (https://www.ncbi.nlm.nih.gov/Structure/cdd/wrpsb.cgi, 19 May 2022). The predicted tail fiber proteins were analyzed for the presence of amino-acid and structural homology with polysaccharide-hydrolyzing enzymes in protein databases by using HHpred (toolkit.tuebingen.mpg.de/tools/hhpred, 19 May 2022), HMMER (https://www.ebi.ac.uk/Tools/hmmer/, 22 May 2022) and InterPro tools (https://www.ebi.ac.uk/interpro/, 19 May 2022). The secondary structures of the protein phage coded were predicted by Phyre2 web server (http://www.sbg.bio.ic.ac.uk/phyre2/phyre2_output/4B1e5088d0c98c2A/summary.html, 9 June 2022). The nucleotide sequence of phage ΦFK1979 was deposited in the GenBank database under the accession number ON146449.

### 2.7. Extraction of bacterial genomic DNA sequencing and bacterial genome comparison

The Bacterial Genomic DNA Kit (Sigma-Aldrich) was used for DNA extraction. The FK1979 wild-type genomic DNA (gDNA) was sequenced by the hybrid method [Nanopore (Oxford Nanopore Technologies, United States) + HiSeq 150 bp paired-end (Illumina, Inc., United States)] and the genomes of the mutants by Illumina HiSeq (Illumina, Inc., United States) at the HuaDa Gene Sci-Tech Company (HuaDa Gene, Beijing, China). We used Snippy^1^ to identify SNPs between the genome sequence of FK1979-WT and phage-resistant mutants. Furthermore, assembled genomes of strains were typed for *Klebsiella* K and O loci by the Kaptive v0.7.3 Web algorithm (https://github.com/katholt/Kaptive, 20 January 2022). The nucleotide sequence of chromosome and plasmid of FK1979 was deposited in the GenBank database under the accession number CP094940 and CP094941.

### 2.8. Host range and efficiency of plating analysis

The host range of phage ΦFK1979 was established by spot test and EOP analysis as previously described (Pertics et al., 2021) on 35 *K. pneumoniae* strains (Table 1). The efficiency of plating (EOP) was calculated as the average PFU on target bacteria divided by the average PFU on host bacteria through three independent assays. The host range of phages isolated in this study was established by spot test on 77 *K. pneumoniae* strains (Table 2).

**Table 1:** Host range of phage ΦFK1979 with *Klebsiella pneumoniae* strains by spot testing and EOP assay.

*Klebsiella pneumoniae* strain	CPS-type	Sequence type	Phage ΦFK1979	
			Spot testing	EOP
FK1979	K2	ST86	+*H*	High
FK4176	K2	ST86	+*H*	High
FK3044	KL2	ST65	+*H*	High
FK4276	K2	ST86	+*H*	High
FK5537	KL2	ST25	+*H*	High
FK6923	KL5	ST1333	-	Inefficient
FK3347	KL54	ST29	-	Inefficient
FK3521	KL54	ST29	-	Inefficient
FK4737	K1	ST367	-	Inefficient
FK3038	K1	ST23	-	Inefficient
NTUH-K2044	K1	ST23	-	Inefficient
FK7757	KL57	ST412	-	Inefficient
FK8123	KL57	ST412	-	Inefficient
FK7521	KL57	ST412	-	Inefficient
FK8716	KL57	ST412	-	Inefficient
FK6449	KL74	ST273	-	Inefficient
FK3518	KL18	ST831	-	Inefficient
FK3068	KL9	ST320	-	Inefficient
FK5119	KL53	ST363	-	Inefficient
FK7685	KL125 (KL114)	ST147	-	Inefficient
FK7117	KL64	ST86	-	Inefficient
FK2682	KL64	ST86	-	Inefficient
FK3092	KL64	ST86	-	Inefficient
FK4603	KL64	ST86	-	Inefficient
FK5164	KL64	ST86	-	Inefficient
FK5222	KL64	ST86	-	Inefficient
FK6716	KL64	ST86	-	Inefficient
MGH 78578	K52	ST38	-	Inefficient
ATCC 700603	K6	ST489	-	Inefficient
ΦFK1979-resistant mutant R2	K2	ST86	-	Inefficient
ΦFK1979-resistant mutant R3	K2	ST86	-	Inefficient
ΦFK1979-resistant mutant R4	K2	ST86	-	Inefficient
ΦFK1979-resistant mutant R5	K2	ST86	-	Inefficient
ΦFK1979-resistant mutant R6	K2	ST86	-	Inefficient
ΦFK1979-resistant mutant R7	K2	ST86	-	Inefficient

+, clear lysis; −, no effect; H, turbid ring around lysis (halo). The phage efficiency was classified as highly productive (efficiency of plating (EOP) ≥ 0.5), moderately productive (0.1 ≤ EOP < 0.5), low productive (0.001 < EOP < 0.1), or inefficient (EOP ≤ 0.001).

**Table 2:** Host range of phages with *Klebsiella pneumoniae* strains by spot testing.

*Klebsiella pneumoniae* strain/isolate	CPS-type	Sequence type	Phage(s) that can lyse the strain
FK1971	KL1	ST23	-
FK2652	KL1	ST23	-
FK3038	KL1	ST23	-
FK3907	KL1	ST23	-
FK4737	KL1	ST367	-
FK7664	KL1	ST23	-
FK8682	KL1	ST23	-
FK3680	KL1 (KL22/37)	ST700	-
FK4956	KL1 (KL22/37)	ST700	-
FK3150	KL10	ST3869-2LV	pR2/pR3/pR4/pR6/pR7
FK7685	KL125 (KL114)	ST147	pR3
FK1689	KL149	ST152	pR2/pR3
FK5223	KL19	ST15	pR3/pR7
FK1979	K2	ST86	pR2/pR3/pR4/pR6/pR7
FK4176	K2	ST86	pR2/pR3/pR4/pR6/pR7
FK3044	KL2	ST65	pR2/pR3/pR4/pR6/pR7
FK4276	K2	ST86	pR2/pR3/pR4/pR6/pR7
FK5537	KL2	ST25	pR2/pR3/pR4/pR6/pR7
FK6048	KL2	ST65	pR2/pR3/pR4/pR6/pR7
FK1819	KL47	ST11	pR3
FK2262	KL47	ST11	pR3
FK2357	KL47	ST11	pR3
FK2686	KL47	ST11	pR3
FK2784	KL47	ST11	-
FK4724	KL47	ST11	pR2/pR3/pR4/pR7
FK6923	KL5	ST1333	-
FK5119	KL53	ST363	pR2/pR3/pR4/pR6/pR7
FK1916	KL54	ST29	-
FK3347	KL54	ST29	pR3
FK3521	KL54	ST29	-
FK3616	KL54	ST29	-
FK7427	KL54	ST29	-
FK8848	KL54	ST29	-
FK7178	KL57	ST412	pR3
FK7757	KL57	ST412	pR3
FK8123	KL57	ST412	pR2/pR3/pR4/pR6/pR7
FK8716	KL57	ST412	pR3
FK7521	KL57 (KL34)	ST2846	-
FK1541	KL63	ST111	-
FK6449	KL74	ST273	pR2/pR3
FK3068	KL9	ST320	pR2/pR3
FK4006	KL9	ST462	pR2/pR3
FK6766	KL64	ST11	pR2/pR3/pR4
FK6777	KL64	ST11	pR2/pR3/pR4
FK6793	KL64	ST11	pR2/pR3/pR4
FK6818	KL64	ST11	pR2/pR3/pR4
FK6824	KL64	ST11	pR2/pR3/pR4
FK6835	KL64	ST11	pR2/pR3/pR4
FK6838	KL64	ST11	pR2/pR3/pR4
FK6839	KL64	ST11	pR2/pR3/pR4
FK6842	KL64	ST11	pR2/pR3/pR4/pR6/pR7
FK6845	KL64	ST11	pR2/pR3/pR4
FK6847	KL64	ST11	pR2/pR3
FK6916	KL64	ST11	pR2/pR3
FK6939	KL64	ST11	pR2/pR3/pR4
FK7009	KL64	ST11	-
FK7014	KL64	ST11	pR2/pR3/pR4/pR6/pR7
FK7040	KL64	ST11	pR2/pR3
FK7072	KL64	ST11	pR2/pR3/pR4
FK7096	KL64	ST11	pR2/pR3/pR4
FK7123	KL64	ST11	pR2/pR3/pR4/pR6/pR7
FK7148	KL64	ST11	pR2/pR3
FK7153	KL64	ST11	pR2/pR3
FK7156	KL64	ST11	pR2/pR3
FK7191	KL64	ST11	pR4
FK7214	KL64	ST11	pR2/pR3/pR4/pR6/pR7
FK7227	KL64	ST11	pR2/pR3
FK7407	KL64	ST11	pR2/pR3
FK2682	KL64	ST11	pR2/pR3/pR4
FK2877	KL64	ST11	pR2/pR3/pR4
FK3092	KL64	ST11	pR2/pR3/pR4
FK3642	KL64	ST11	pR2/pR3/pR4
FK4603	KL64	ST11	pR2/pR3/pR4
FK5164	KL64	ST11	pR2/pR3/pR4
FK5222	KL64	ST11	pR2/pR3/pR4
FK6716	KL64	ST11	pR2/pR3/pR4
FK7117	KL64	ST11	pR2/pR3/pR4

-, indicates none out of phages pR2/pR3/pR4/pR6/pR7 was sensitive to the corresponding *K. pneumoniae* isolate.

### 2.9. Isolation and confirmation of phage-resistant mutant

As described in a previous study (Liu M. et al., 2022) with some modification, 10 μL undiluted phage ΦFK1979 was spotted on the lawn of *K. pneumoniae* FK1979. Phage-resistant mutants were obtained after 24 h by picking the bacterial colonies growing in the middle of lysis zones and streaking them onto LB **agar** plates. Then, we mixed these isolates with phage ΦFK1979 and cultured them in LB broth for 6 h at 37°C until the possible sensitive bacteria are lysed. The cultures were then plated on LB **agar** to produce individual colonies. A single colony was isolated from these plates and purified by three rounds of subculture. The phage-resistant phenotype was confirmed using spot tests and EOP assays as described above. The resistant mutants were 10 successive passages on LB agar to confirm the stability of the resistant phenotype. Species were confirmed by genome sequencing.

### 2.10. Phage adsorption rate measurement

Add 10 μL of 1.5 × 10^8^ PFU/mL phages (initial phage titers determined by double-layer plate method) to 2 mL of subcultured end-logarithmic growth bacteria (the concentration of 1 × 10^9^ CFU/mL bacteria calculated by CFU counting), and the mixture was incubated for 10 min at 37°C 180 r/min. The mixture was centrifuged for 5 min (4°C, 12,000 × g), and the supernatant was filtered with a 0.22 μm filter and was diluted by 10-fold serial dilution. The double-layer agar plate method was used to determine the residual phage titer in the supernatant. The phage adsorption rate was calculated as phage adsorption efficiency = [(Initial titer of phage-residual titer in the supernatant)/Initial titer] × 100%.

### 2.11. Assessment of capsule production and hypermucoviscosity

The string test was a qualitative assay for hypermucoviscosity (HMV; >5 mm). HMV was assessed by measuring the optical density at 600 nm (OD600) of culture supernatants following low-speed centrifugation (1,000 × g for 5 min). SEM (SU-8010, Hitachi, Tokyo, Japan) was performed for analysis of the surface morphology of the strains. Acidic polysaccharides were extracted and measured to quantify capsular polysaccharides as described previously (Liu S. et al., 2022). Briefly, 500 μL of the bacterial culture of log-phase was mixed with 100 μL of 1% ZWITTERGENT 3–14 detergent in 100 mM citric acid, followed by incubation at 50°C for 20 min. The cells were then pelleted (14,000 × g for 2 min). 300 μL of the supernatant were aspirated and mixed with 1.2 mL of absolute ethanol, incubated at 4°C for 20 min, and then centrifuged for 5 min at 14,000 × g. The pellet was dried and resuspended in 200 μL of distilled water. Then 1.2 mL of 12.5 mM sodium tetraborate in sulfuric acid was added and incubated for 5 min at 100°C, followed by incubation on ice for 10 min. Thereafter, a 20-μL volume of 0.15% 3-phenyl phenol in 0.5% NaOH was added. After a 5-min incubation at room temperature, the absorbance at 520 nm was measured. The glucuronic acid content was then determined from a standard curve of glucuronic acid and expressed as μg/OD unit.

### 2.12. Bacterial growth curve determination

The growth kinetics of the strains was determined by monitoring culture optical density (OD at 600 nm) for 24 h at 37°C. Bacterial cultures with the starting density of 5 × 10^5^ CFU/mL were grown in a flat-bottomed 96-well microplate (Corning, United States), with a shaking cycle every 30 min when OD_600_ was measured by a microplate reader (Multiskan FC).

### 2.13. Serum resistance assay

The resistance of bacteria to serum bactericidal activity was determined in pooled normal human serum. Briefly, ~1 × 10^6^ CFU of log-phase bacteria were suspended in PBS and mixed at a 1:3 v/v ratio with serum. The mixture in a final volume of 1 mL was incubated for 3 h at 37°C, and at 0, 1, 2, and 3 h intervals; then, 100 μL aliquots were removed, diluted, and cultured on LB agar for colony enumeration.

### 2.14. Biofilm formation assays

24 h biofilm formation on polystyrene 96-well plates (Corning, United States) was assessed using crystal violet (Solarbio, China) staining and spectrophotometry under the OD_570nm_ with a microplate reader (Multiskan FC) as previously described (Zheng et al., 2018).

### 2.15. Cell assays

Human lung epithelial cells A549 maintained in RPMI-1640 medium and murine macrophage cells RAW 264.7 (Procell Life Science and Technology Co., Ltd., China) in DMEM medium, respectively, were supplemented with 10% heat-inactivated fetal bovine serum (HIFBS; GIBCO, Life Technologies) and 1× antibiotic–antimycotic solution (GIBCO, Life Technologies, USA) at 37°C in a 5% CO_2_. For the adhesion experiment, A549 or RAW 264.7 cells were seeded at an initial density of 5 × 10^5^ cells per well in 24-well flat-bottom microplates (Nest, United States). Subsequently, bacteria in triplicate were added (MOI = 20) and co-incubated for 3 h. To evaluate the invasive abilities of strains on cells, 1 mL of 100 μg/mL gentamicin (Kangtai, China) was added to kill the extracellular and adherent bacteria after incubation for 3 h. After washing with PBS five times, the bacteria were released with 500 μL of 0.1% Triton X-100 solution. The bacteria were 10-fold serial diluted and plated onto LB agar for colony count.

### 2.16. *Galleria mellonella* infection assay

The *G. mellonella* model was used to study the *in vivo* virulence of bacteria. Groups of 10 *G. mellonella* larvae (Tianjin, China) were subjected to an injection of 10^5^ CFU (± 0.5 log) of *K. pneumoniae*. The blank control group was injected with 10 μL PBS. The survival rates of larvae were recorded for 72 h post-injection at 12 h intervals.

### 2.17. Mice infections

The survival curves of mice infected with each bacterium were plotted to further determine the *in vivo* virulence of bacteria. 10 BALB/c mice (Zhejiang Vital River Laboratory Animal Technology Co., Ltd., China) were used as a sample population for each tested strain. Each strain was adjusted to 0.5 McF and mice were infected with 0.2 mL (~3 × 10^7^ CFU) of different *K. pneumoniae* strains intraperitoneally. The previously reported hvKP strain NTUH-K2044, cKP strain ATCC700603, and saline were applied as the controls. The survival rates of the inoculated mice were recorded for 7 days post-injection at 12 h intervals. The mice used in this study were approved by the Laboratory Animal Ethics Committee of the First Affiliated Hospital of Wenzhou Medical University (WYYY-AEC-2022-047).

### 2.18. RNA extraction and transcriptional expression of genes by quantitative real-time PCR

The expression levels of polysaccharides biosynthesis relevant genes (*wcaJ*, *wzi*, *galF, galU, wbbM*, *wzm, wzt, rfaH, waaE, wcaG, rcsA*, mutant genes encoding polysaccharide deacetylase family protein (pdfp) and sugar glycosyltransferase (sgtr) discovered in SNP analysis above) were detected by RT-qPCR. Overnight bacterial cultures of the FK1979-WT strain and its mutants were incubated with ΦFK1979 for 10 min. Subsequently, total RNA was extracted by Trizol method. The quantitative real-time PCR (RT-qPCR) primers are listed in Supplementary Table S1. FK1979-WT served as the control group and phage-resistant mutants as the experimental groups. The relative expression of the indicated genes was calculated by the 2^−ΔΔCt^ method.

### 2.19. Gene knockout and complementation

Genes deletions in *K. pneumoniae* FK1979 were performed by the lambda-Red knockout system. Briefly, the FRT-flanked kanamycin resistance cassette was amplified from the pKD4 plasmid using a homologous region primer. The pACBSR-Hyg plasmid was introduced into FK1979-WT by electroporation for recombination. The knockout cassette was transformed into FK1979-WT-pACBSR-Hyg. Correct transformants were verified by colony PCR and Sanger sequencing. The resistance marker was removed by pCP20-Chl plasmid.

The amplified target gene (*galU*) and its promoter were cloned into the pACYC184-Chl plasmid, which was digested with the BamHl enzyme (Takara, Dalian, China). The recombinant plasmid was introduced to Φ-R mut5 strain via electronical transformation. The empty vector (pACYC184-Chl) was introduced to the same strain as a blank control. The primers, plasmids and strains used are listed in Supplementary Tables S1, S2.

### 2.20. Data availability

The genetic sequences acquired during this study have been deposited into the National Center for Biotechnology Information database as a BioProject under accession number PRJNA822025. The project contains data for *K. pneumoniae* strains FK1979 (accession number SAMN27162961) and phage-resistant strains Φ-R mut 2–7 (accession number SAMN32741764, SAMN32741765, SAMN32741766, SAMN32741767, SAMN32741768, SAMN32741769). The genomes of phages were submitted on GenBank (Accession number shown in Table 3). Source data are provided in this paper.

**Table 3:** Information of phages of *Klebsiella pneumoniae* strains isolated and characterized in this study.

Name	DNA	Genome size (bp)	GC content %	ORF number	Genebank accession
ΦFK1979	Linear double-stranded	43,644	53.84	53	ON146449
pR2	Circular double-stranded	178,906	41.46	283	OR138016
pR3	Circular double-stranded	169,297	40.75	287	OR138018
pR4	Circular double-stranded	175,661	41.92	273	OR138017
pR6	Circular double-stranded	178,070	41.73	282	OR138015
pR7	Linear double-stranded	176,880	41.92	275	OP684128

### 2.21. Statistical analysis

Each experiment was performed in triplicate. Statistical significance of the differences in biofilm biomass and bacterial mucoviscosity between the wild-type control group and the mutant groups was analyzed by unpaired *t* tests, two-tailed; a Mann–Whitney test for CFU or PFU difference. A repeated measures analysis of variance (ANOVA) was used to assess changes in continuous repeated measures variables over time, including temporal killing assay of phage, bacterial growth, and serum resistance. Survival curves were analyzed via a Kaplan–Meier analysis and a log-rank test. ^***^p < 0.001; ^****^p < 0.0001; n.s., no significance. The statistical analyses were performed using the SPSS and GraphPad Prism 8.03 software.

## 3. Results

### 3.1. Isolation and characterization of phage ΦFK1979

**Figure 1:**
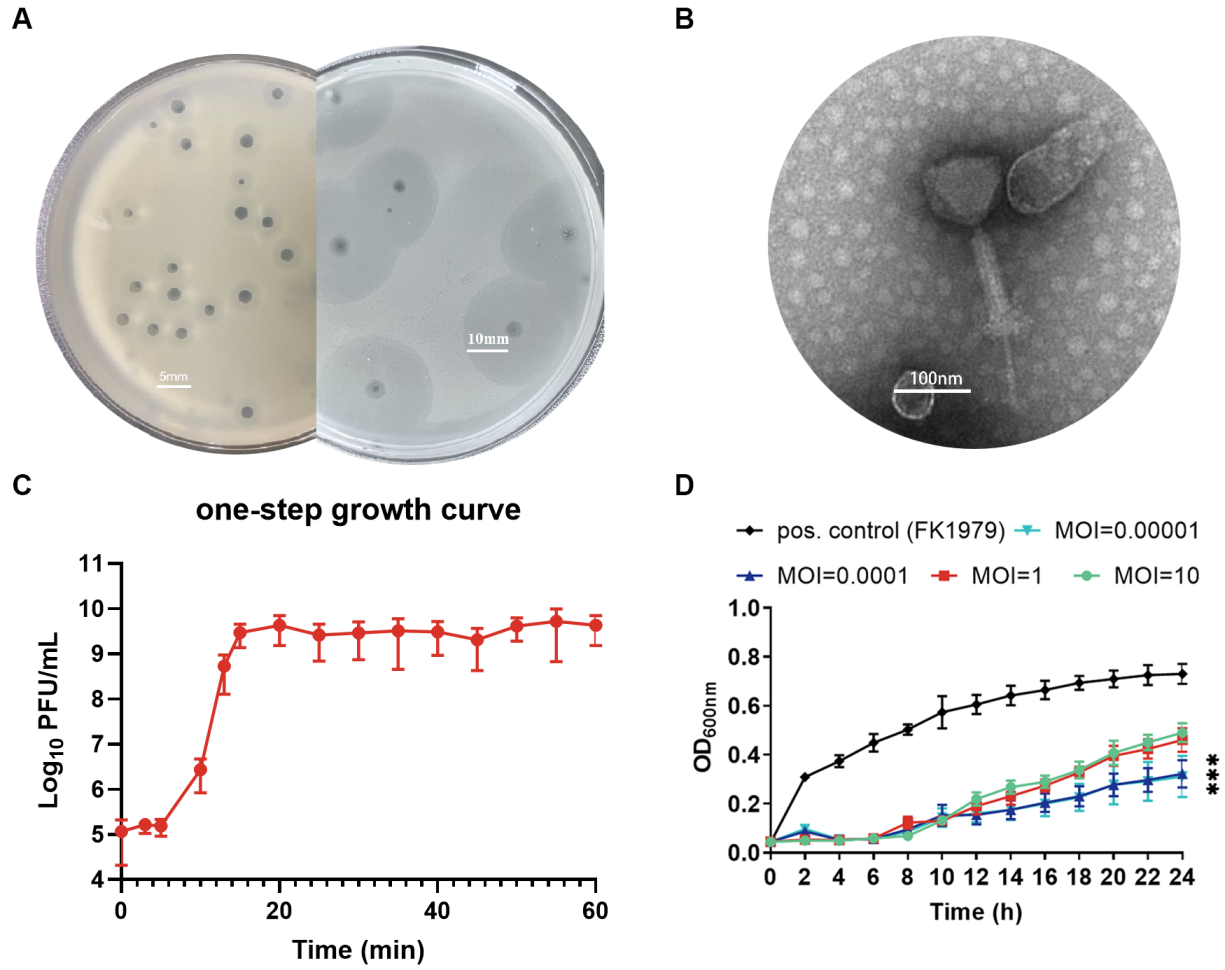
Characteristics of phage ΦFK1979. **(A)** Plaque morphology formed by *Klebsiella pneumoniae* phage ΦFK1979 using overlay agar plate with *Klebsiella pneumoniae* FK1979-WT lawn. Scale bars represent 5 mm and 10 mm, respectively. represents 5 mm. **(B)** Transmission electron micrograph image of phage ΦFK1979. Scale bar represents 100 nm. **(C)** One-step growth curve of the phage ΦFK1979. Phage-forming plaque-forming units (PFU) at different time-point within 60 min are shown. **(D)** Temporal killing assay of phage ΦFK1979 against *K. pneumoniae* FK1979. Overnight bacterial cultures of FK1979 (10^3^ CFU/mL) were infected with phages with different multiplicity of infection (MOI). Bacterial cultures without phage treatment were used as a positive control (◆). Each data point is the average of the three experiments. Standard error (± SEM) appears as a vertical line. A repeated measures analysis of variance (ANOVA) was used to assess the statistical significance.

Phage ΦFK1979 was isolated from hospital sewage using the clinical isolate of *K. pneumoniae* FK1979 as a bait bacterial host. Purified phage ΦFK1979 formed plaques displaying a clear center (1–2.5 mm in diameter) with a turbid ring (3.5–5 mm in diameter) surrounding it on bacterial moss of its host *K. pneumoniae* FK1979. After prolonged culture for 48 h, a halo of 15–20 mm in diameter was observed, indicating the lytic nature against *K. pneumoniae* FK1979 and the polysaccharide depolymerase production of ΦFK1979 (Latka et al., 2017; Figure 1A). TEM image of phage ΦFK1979 indicated a tailed phage (Caudovirales) morphotype, with a 90–100 nm icosahedral head and a ~210 nm-long tail, which indicated it possesses a polysaccharide depolymerase protein at the end of its tail (Olson et al., 2021; Figure 1B). The one-step growth curve indicated that ΦFK1979 replicates quickly with a short latency of ~5 min, a burst size of 292 PFU per bacterium and a lytic cycle length of 20 min on its host strain FK1979 (Figure 1C). Phage ΦFK1979 efficiently inhibited the growth of *K. pneumoniae* FK1979 for 24 h with relatively high MOIs of 10 and 1, as well as low MOIs of 0.0001 and 0.00001, but the killing curve at the MOI of 0.0001 exhibited the most significant inhibition of bacterial growth, indicating that the OMOI was 0.0001 (Figure 1D). The host range of ΦFK1979 was measured with 29 clinically isolated and reference *K. pneumoniae* strains with documented capsular types (K1, K2, K5, K54, K57, KL74, KL18, KL9, KL53, KL125, KL64, K52, and K6) by spot test and EOP assays, which showed that ΦFK1979 formed clear plaques with high efficiency on its host FK1979-WT and other K2 capsular-types strains from different sources, but not on others, indicating that phage ΦFK1979 was relatively specific for K2 capsular-types strains (Table 1).

### 3.2. Genomic and phylogenetic characteristics of phage ΦFK1979

**Figure 2:**
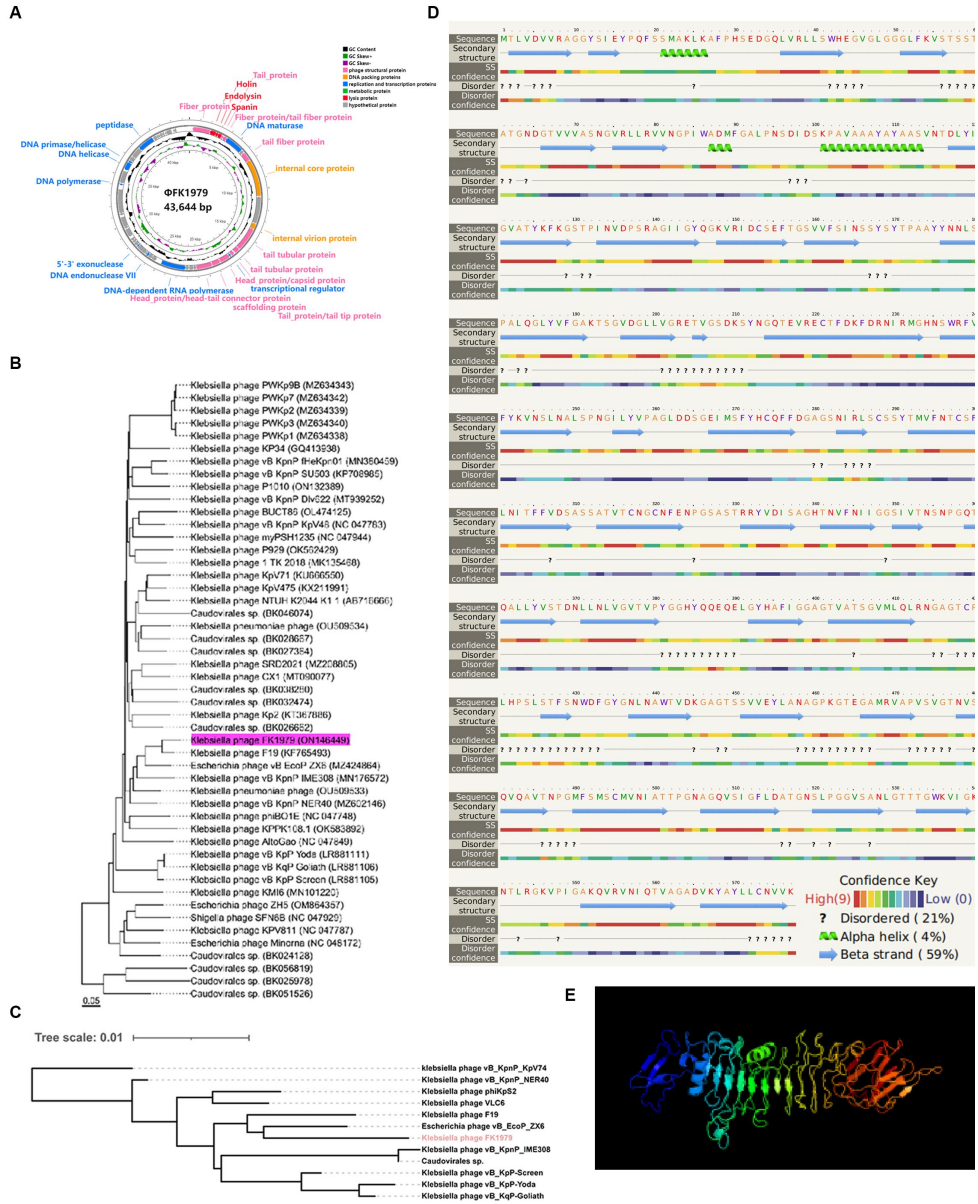
Genomic and phylogenetic characteristics of phage ΦFK1979. **(A)** Genome map of the bacteriophage ΦFK1979. The predicted ORFs and direction of transcription are indicated by arrows. Genes are colored based on function classification. Pink represents phage structural protein, blue represents phage replication and transcription proteins, DNA packing proteins are colored orange, phage metabolic proteins are colored green and lysis proteins are colored red. **(B)** Phylogenetic relationship of phage. The whole genome phylogenetic tree was created using the Genome-BLAST Distance Phylogeny method in VICTOR. **(C)** Neighbor-joining phylogenetic tree based on phage tail fiber protein (encoded by ORF1 in ΦFK1979) similarity between the phage ΦFK1979 and other 11 similar phages publicly available at NCBI. **(D)** Predicted secondary structure and **(E)** three-dimensional structure of the protein encoded by ORF1 of ΦFK1979 by Phyre2 web server. NORMAL mode. Confidence: 99.9% and Coverage: 81% in the model. 468 residues (81% of sequence) have been modeled with 99.9% confidence by the single highest-scoring template. Model dimensions (Å): X:83.801, Y: 84.704, Z: 135.248. Image colored by rainbow N → C terminus.

DNA sequencing showed that the ΦFK1979 genome is a linear double-stranded DNA of 43,644 bp with a 53.84% G + C content (Genbank accession ON146449). Phage ΦFK1979 was predicted to contain 57 Open reading frames (ORF)s. Most of the ORFs are functionally unknown, and 24 ORFs can be categorized into several functional modules, including phage structural protein, phage replication and transcription proteins, DNA packing proteins, phage metabolic protein and lysis proteins (Figure 2A; Supplementary Table S3). No virulence, lysogenic, antimicrobial resistance, or integrase/repressor genes were included. PHACTS Lytic Score of 0.851 indicating a < 20% probability of exhibiting temperate behavior revealed that ΦFK1979 is likely a lytic phage. These results strongly suggested that ΦFK1979 is a safe candidate for phage therapy. Genome-BLAST analysis showed that ΦFK1979 is closely related to the *Klebsiella* phage F19 (97.75% identity, YP_009006074.1), which belongs to the Drulisvirus genus of Slopekvirinae subfamily of Autographiviridae family of Caudoviricetes order (Figure 2B). Identify Conserved Domains of NCBI revealed an “N-terminal and middle domains of tail spike protein in *Acinetobacter* bacteriophages” in ORF1 (UPW35138.1) and an “N-terminal phage_T7_tail fiber protein” in ORF9 (UPW35146.1). The neighbor-joining tree showed that ORF1 from phage ΦFK1979 had a high similarity to the tail fiber proteins expressed by *Escherichia* phage vB_EcoP_ZX6 (97.75% identity, QXO10373.1) and *Klebsiella* phage F19 (97.75% identity, YP_009006074.1; Figure 2C). ORF1 showed a similarity to the polysaccharide depolymerase and hydrolase active domains of tail spike proteins of multiple phages according to the HHpred search, and most of the hits in HMMER search were also phage tail fiber proteins. The secondary structure of the protein prediction showed that ORF1 contains multiple alpha-helix domains and beta-plated sheets which usually represent the functional domains in proteins (Figure 2D). The predicted three-dimensional structure of the protein encoded by ORF1 (Figure 2E) also showed that it had a high similarity to the structures of polysaccharide depolymerase of other well-documented phages. According to these results, the tail fiber protein encoded by ORF1 of ΦFK1979 is most likely a phage polysaccharide depolymerase (Cai et al., 2019; Thiry et al., 2019; Liu et al., 2020).

### 3.3. Phenotype and genomic characteristics of host *Klebsiella pneumoniae* FK1979

*Klebsiella pneumoniae* FK1979 exhibited a large, protruding, smooth and sticky colony morphology on the blood agar plate similar to reference hvKP NUTH-K2044 but completely different from classic *K. pneumoniae* ATCC 700603. Congo red agar assay, in which hvKp is usually blackening due to the EPS production, was observed positive for FK1979 comparable with hvKP NUTH-K2044 (Supplementary Figure S1A). The string assay showed that FK1979 had a significant hypermucoviscosity phenotype compared with the same hypermucoviscos NUTH-K2044 and the string-negative ATCC 700603 (Supplementary Figure S1B). The whole genome sequence and comparative analysis of FK1979 showed that it possessed an almost identical genome to referenced hvKP CG43, NUTH-K2044, and ATCC43816 (Short et al., 2020; sequence identity 99.98%–100% with 99%–100% query cover, BLASTN; Supplementary Figure S1C) and contained the same set of a large hypervirulent plasmid highly similar to pK2044 (NC_006625.1) and pLVPK (NC_005249.1; Supplementary Figure S1D). FK1979 belonged to the K2 capsule type and O1v1 O antigen serotype, as well as ST86 sequence type hvKP through K/O loci and MLST analysis. All in all, the phenotypic and genomic characteristics of FK1979 illustrated that FK1979 produces a thick capsule and is a typical hypermucoviscosity and hypervirulent K2, ST86 *K. pneumoniae* strain.

### 3.4. The emergence of phage-resistant mutants

The phage resistance formation was verified by a spot test, an EOP assay, and an adsorption assay for the sensitivity of the wild-type strain and resistant mutants to phage ΦFK1979. As shown in the spot test and EOP assays, the absence of plaque formation on the six phage-resistant mutants (Φ-R mut2-7) was indicative of phage resistance (Supplementary Figure S2A; Table 1). The adsorption assay of phage ΦFK1979 onto FK1979-WT and phage-resistant mutants showed that the absorption capability of mutants was significantly lower than that of the FK1979-WT strain, indicating the development of resistance to phage ΦFK1979 during adsorption (Supplementary Figure S2B; the results of representative Φ-R mut2 and Φ-R mut5 were shown). The colony morphology of strains changed obviously after phage resistance formed at first isolation (Supplementary Figure S2C). And these results remained the same after 10 serial passages of the strains, indicating the stability of the resistance phenotype over multiple generations (Supplementary Figure S2D). Taken together, all these observations indicated that after ΦFK1979-driven selection, the phage-resistant mutants derived from FK1979 rapidly and stably appeared.

### 3.5. Capsule deficiency contributes to phage resistance

**Figure 3:**
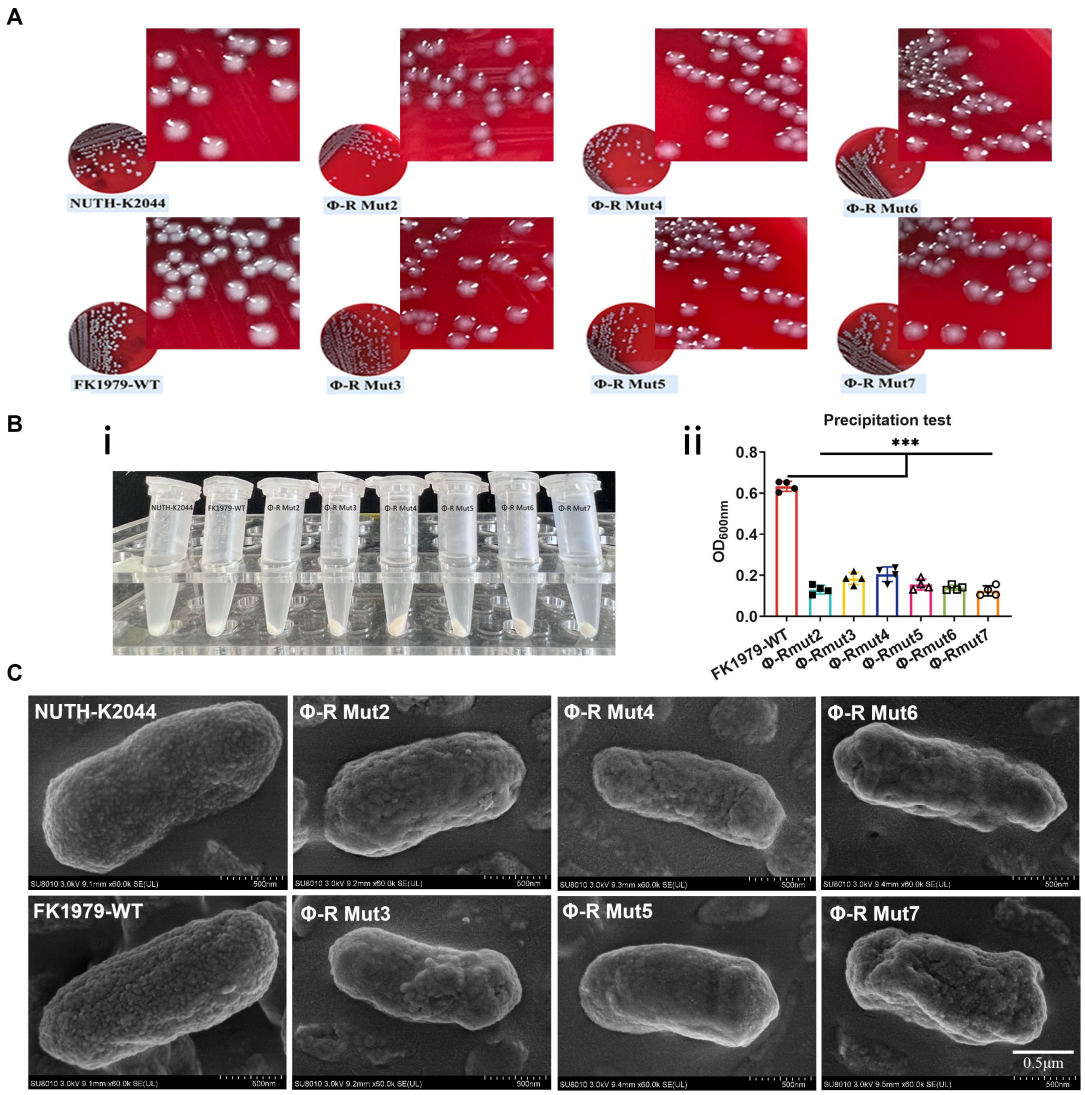
Phage-resistant mutants were characterized by a deficiency of the capsule/mucoviscosity of *K. pneumoniae*. **(A)** Colonies morphology comparison of *K. pneumoniae* strains. **(B)** Bacterial mucoviscosity assay. **(i)** Image of bacterial culture after centrifugation. **(ii)** The absorbance of the supernatant was determined at 600 nm using a spectrophotometer. Statistical significance of the differences between the WT control group and the mutant groups was analyzed by unpaired *t* tests, two-tailed; ^***^p < 0.001. **(C)** Cell surface appearance visualized by SEM suggests reduced capsule production in the mutants Φ-R mut2-7.

The capsule roughness has been attributed to the precipitation of polysaccharides and their deposition onto the surface of bacteria. The colony morphology of the phage-resistant strains exhibited dry, rough, transparent, and flat, while their parental strain FK1979-WT was mucoid, moist, protruding and sticky (Figure 3A). The effect of phage resistance on the CPS production was tested by centrifugal precipitation test. After centrifugation, all six mutants pelleted tightly, while FK1979-WT and positive control strain hvKP NUTH-K2044 remained turbid. There were more suspended bacteria for the wild-type than for the phage-resistant mutants (Figure 3Bi). The OD600 of supernatant from the positive control hvKP NUTH-K2044 or FK1979-WT sample was higher than that from the phage-resistant strains (Figure 3Bii). The result indicated that the mucoviscosity or CPS production of the phage-resistant mutants faded compared with FK1979-WT because highly mucoviscous bacteria are difficult to pellet by low-speed centrifugation. SEM showed that the mutants possessed a smooth surface, significantly different from that of FK1979-WT which exhibited a thick and rough surface (Figure 3C). According to these results, we confirmed that the phage-resistant mutants had less exopolysaccharide surrounding themselves than the wild-type, and capsule deficiency contributed to phage resistance.

### 3.6. The mechanisms by which phage resistance evolves

**Figure 4:**
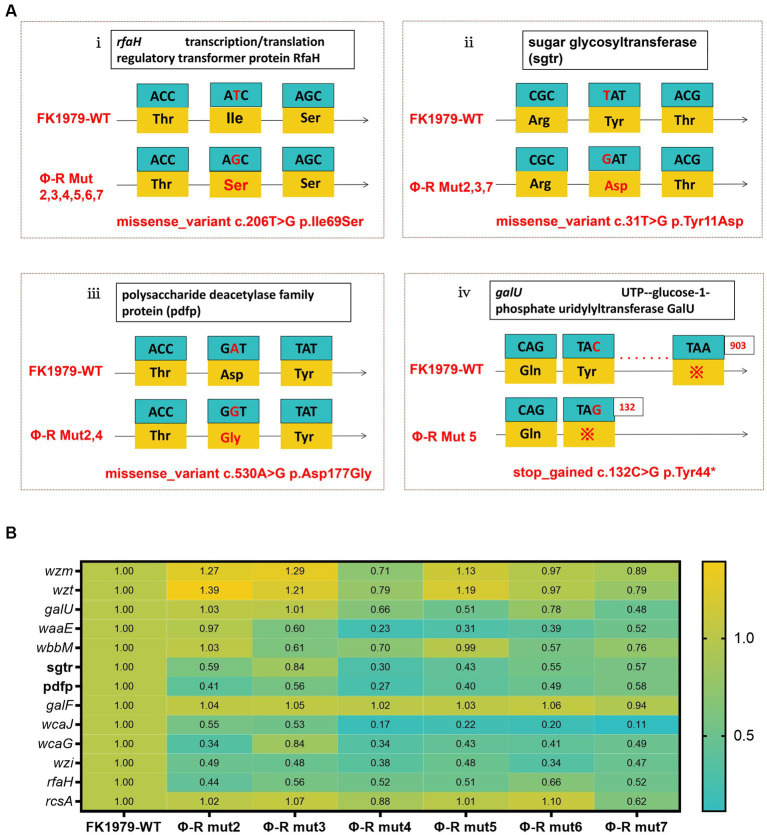
The mechanisms by which phage resistance evolves. **(A)** Schematic representation of genetic alteration in *K. pneumoniae* FK1979 and ΦFK1979-resistant mutants. Codons and amino acids are indicated in the box, and arrows indicate the transcription orientation. **(i)** missense mutation in anti-antiterminator RfaH encoding gene *rfaH*; **(ii)** missense mutation in a sugar glycosyltransferase encoding gene; **(iii)** missense mutation in a polysaccharide deacetylase family protein-encoding gene and **(iv)** stop-gained mutation in *galU*. **(B)** The heatmap of transcript analysis of *K. pneumoniae* polysaccharides biosynthesis relevant genes carried out by RT-qPCR with FK1979-WT strain as a control group and its phage-resistant mutants Φ-R mut2/3/4/5/6/7 as the experimental group. The expression of the housekeeping gene *rpoB* was used for data normalization, and the relative expression levels were estimated by setting the transcript level in FK1979-WT as 1. The relative expression levels value of >2 and <0.5 is regarded as up-regulated and down-regulated significantly, respectively, compared to FK1979-WT control strain. Each data point is the mean of three experiments with a ± standard error (SEM) shown as a vertical line.

To elucidate the mechanisms by which phage resistance evolves, we first compared the genomes of phage-resistant strains against the wild type FK1979-WT, and identified that there was a missense mutation in *rfaH* gene encoding transcription/translation regulatory transformer protein RfaH or being called the transcription antiterminator in all the six mutants (Figure 4Ai). Further, there was a missense mutation in a gene encoding sugar glycosyltransferase in three out of the six mutants, including Φ-R mut2/3/7 (Figure 4Aii) and a gene encoding polysaccharide deacetylase family protein in two out of the six mutants, including Φ-R mut2/4 (Figure 4Aiii). In addition, there was a stop-gain mutation in *galU* gene encoding UTP—glucose-1-phosphate uridylyltransferase galU resulting in early termination of gene transcription and translation and cannot fully express the protein in Φ-R mut5 (Figure 4Aiv). These results verified the interruption of genes responsible for the synthesis of CPS or LPS as the ΦFK1979-resistance mechanism. Secondly, the expressions of polysaccharides biosynthesis relevant genes in FK1979-WT and its phage-resistant derivative were detected by RT-qPCR. As shown in Figure 4B, the expression levels of polysaccharides biosynthesis relevant genes [*wcaJ*, *wzi*, *galF, galU, wbbM*, *wzm, wzt, rfaH, waaE, wcaG, rcsA*, genes encoding polysaccharide deacetylase family protein (pdfp) and sugar glycosyltransferase (sgtr)] were dramatically down-regulated in phage-resistant mutants compared with FK1979-WT. These results indicated that the down-expression of these genes may have a role in the reduced expression of capsular which acts as the most direct primary adsorption receptor of phage ΦFK1979, thereby promoting resistance to phage ΦFK1979 of FK1979-WT.

**Figure 5:**
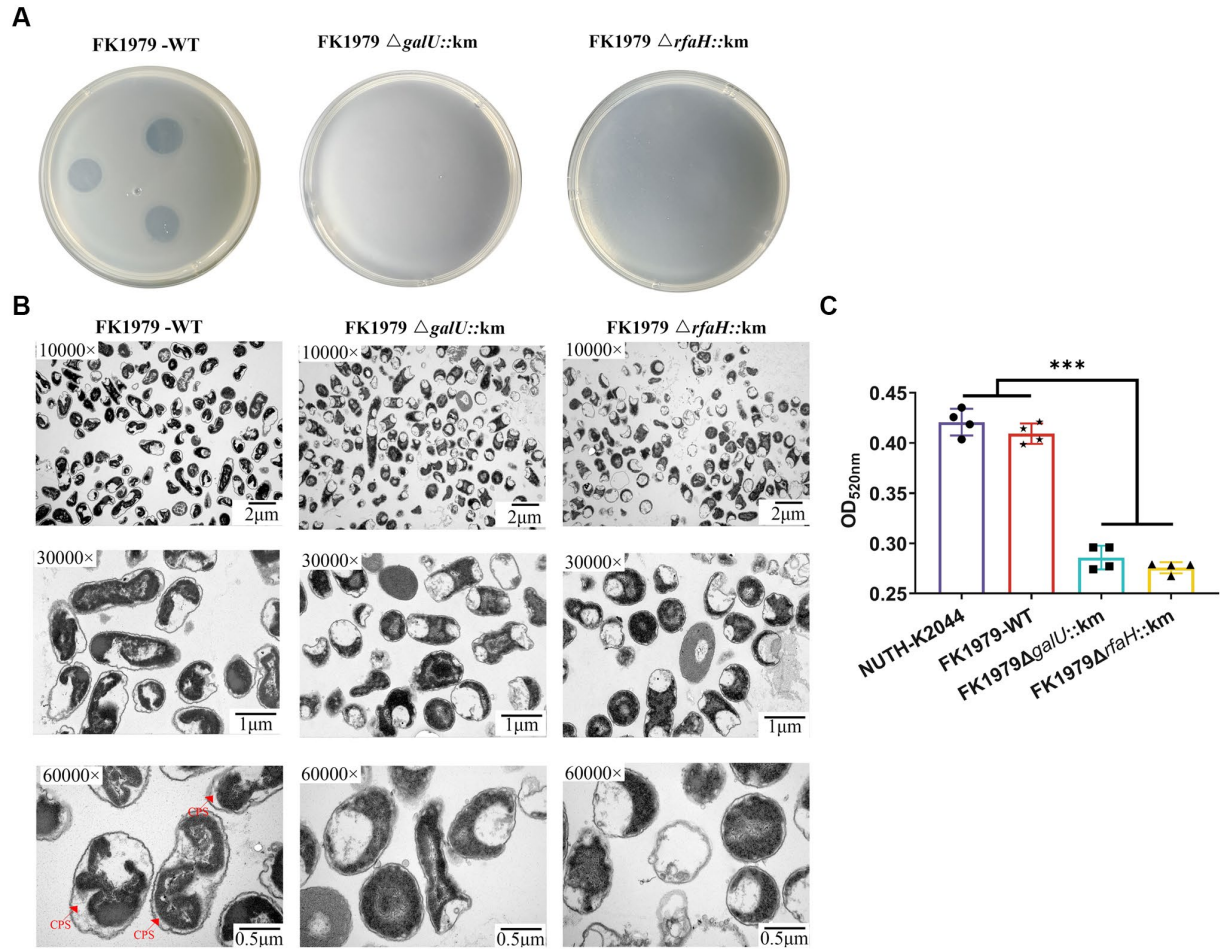
Phage sensitivity and capsule layer of the △*galU* or △*rfaH* mutants. **(A)** Spot test assay of phage on the parental *Klebsiella pneumoniae* strain FK1979-WT and its △*galU* or △*rfaH* mutants. **(B)** Observation of the capsule production of FK1979-WT, △*galU* or △*rfaH* mutants by TEM. For every isolate, one representative image from six images obtained from one section with three magnifications is shown. **(C)** The quantitative experiment of the capsule production of FK1979-WT, △*galU* or △*rfaH* mutants. The previously reported hvKP strain NTUH-K2044 was used as a positive control of capsule high production. ^***^p < 0.001 represents comparison with FK1979-WT or NTUH-K2044.

**Figure 6:**
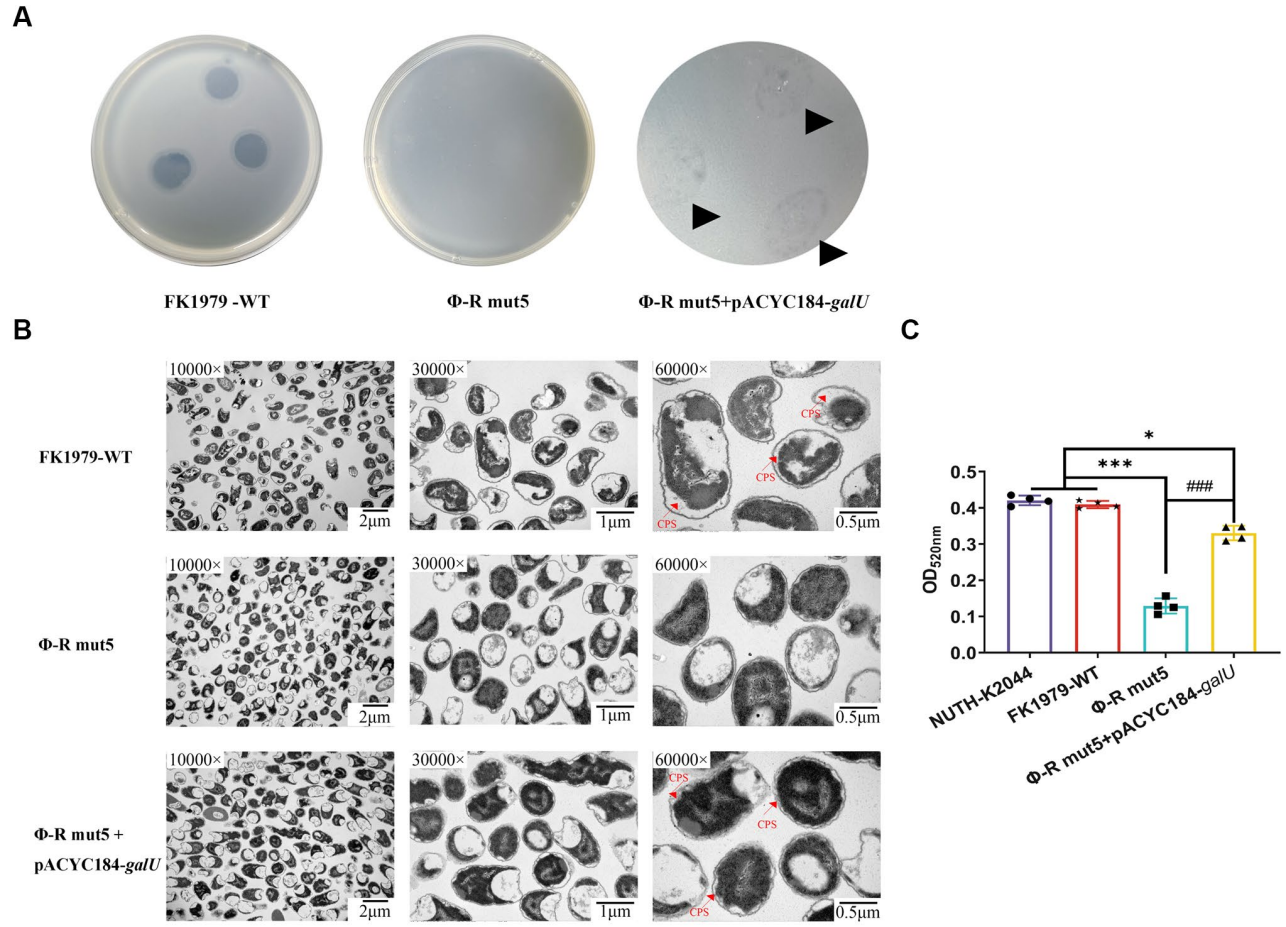
Phage sensitivity and capsule production of the phage-resistant mutant Φ-R mut5 restored by *galU* complementation. **(A)** Spot test assay of phage on the parental *Klebsiella pneumoniae* strain FK1979-WT and its derived mutant Φ-R mut5. **(B)** TEM of WT, phage-resistant, and complementation strain. For every isolate, one representative image from six images obtained from one section with three magnifications is shown. **(C)** The quantitative experiment of the capsule production of FK1979-WT, Φ-R mut5, and *galU* complemented strain. The previously reported hvKP strain NTUH-K2044 was used as a positive control of capsule high production. ^*^p < 0.05 and ^***^p < 0.001 represent comparison with FK1979-WT; ^###^*p* < 0.001 represents comparison with Φ-R mut5.

To demonstrate that the identified mutations were responsible for the phage-resistant phenotype, we disrupted the *galU* gene and the *rfaH* gene, respectively, in FK1979-WT. The FK1979Δ*galU* and FK1979Δ*rfaH* strains were completely resistant to phage ΦFK1979 (Figure 5A). TEM analysis suggested that the surface of the wild-type included a capsule, whereas there was no capsule on the surface of the FK1979Δ*galU* and FK1979Δ*rfaH* strains (Figure 5B). Capsule quantification showed that the production of capsules of gene knocked-out strains was significantly decreased than that of the wild-type (Figure 5C). Through *galU* gene complementation of FK1979-WT into Φ-R mut5 with *galU* mutant, adsorption was partly reinstated in the plasmid-complemented strain (Figure 6A). We also observed a marked increase in the thickness of the capsule of the *galU* gene complemented strain, similar to the wild-type, contrary to the no-capsule Φ-R mut5 strain in TEM micrographs (Figure 6B). The capsule quantitative experiment revealed that the production of the capsule of the *galU* gene complemented strain was dramatically elevated than that of the Φ-R mut5 strain (Figure 6C). Thus, CPS is the receptor for phage ΦFK1979 and phage resistance arose through alteration or loss of CPS, leading to inhibition of phage adsorption.

### 3.7. Fitness costs of phage-resistant mutants

**Figure 7:**
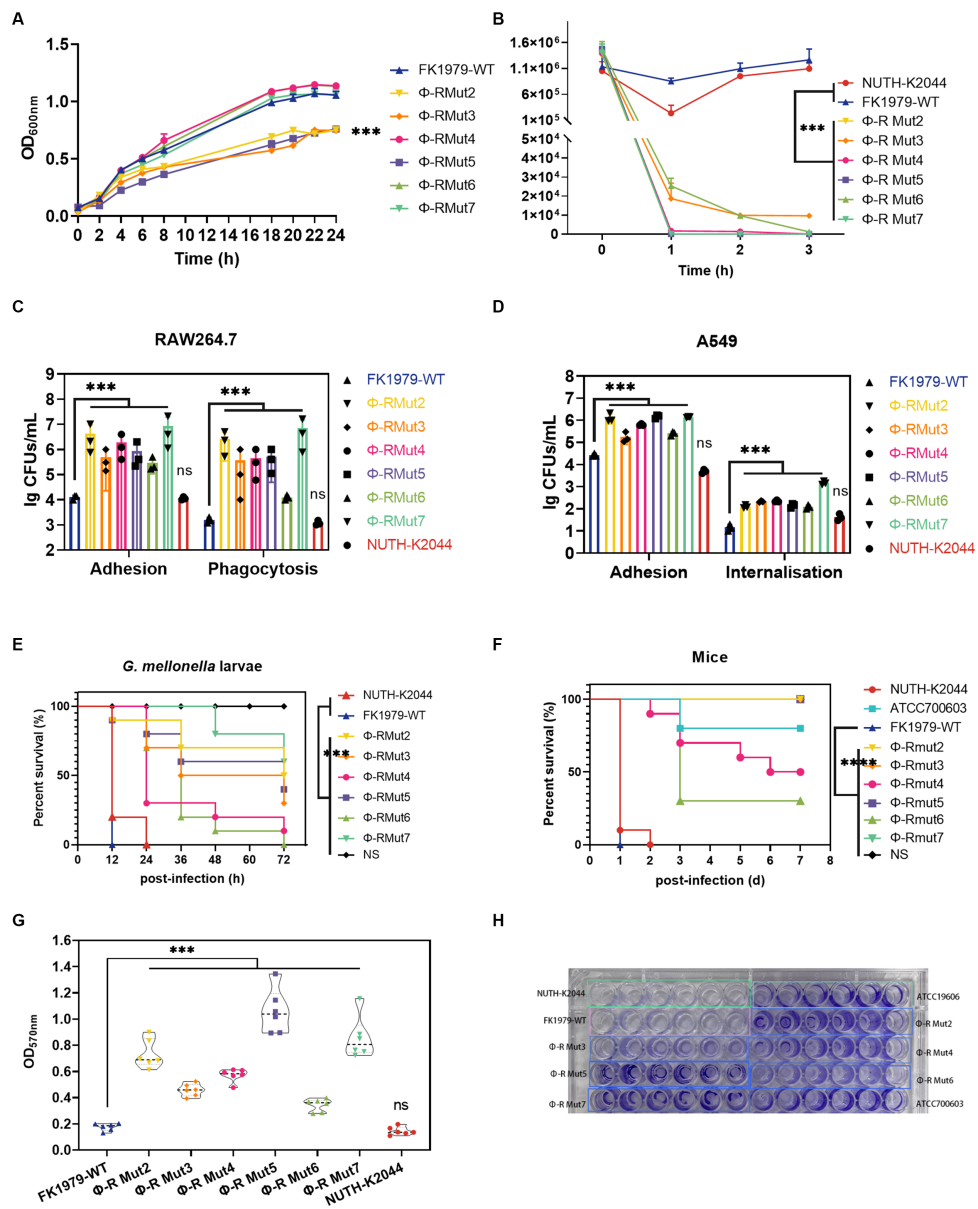
Fitness costs of phage-resistant mutants. **(A)** Growth dynamic curve of FK1979-WT and phage resistant mutants Φ-R mut2-7 for 24 h. The growth abilities of phage-resistant strains Φ-R mut2/3/5 were dramatically decreased compared with wild-type strain FK1979-WT and Φ-R mut4/6/7. Data are mean ± SEM (*n* = 3). **(B)** Human serum killing assay. Fresh pooled healthy human serum was inoculated with 10^6^ CFU/mL of *Klebsiella pneumoniae*, and the change in bacterial load was measured at 1, 2, and 3 h. Wild-type strain FK1979-WT and the positive control of virulence HvKP NUTH-K2044 grew in serum whereas phage-resistant strains Φ-R mut2-7 were rapidly killed. Data are mean ± SEM (*n* = 3). **(C)** (i) Attachment of strains to murine macrophage RAW 264.7 cell line. (ii) Phagocytosis by the RAW 264.7. Infection of the RAW 264.7 cell line was carried out with *K. pneumoniae* FK1979-WT or Φ-R mut2-7 with MOI = 1:20 for 3 h. Phagocytosis different from adhesion was adding gentamicin to kill all extracellular bacteria. Φ-R mut2-7 were more prone to adhere to or be phagocytized by RAW264.7 than wild-type strain FK1979-WT and HvKP NUTH-K2044. **(D)** (i) Adhesion of strains to A549 cells. (ii) Internalization by A549 cells. Infection of the A549 cell was carried out with *K. pneumoniae* FK1979-WT or Φ-R mut2-7 with MOI = 1:20 for 3 h. Bacterial internalization was assessed by the gentamicin protection assay. Φ-R mut2-7 were more susceptible to adhesion to and internalization by A549 than *K. pneumoniae* FK1979-WT and HvKP NUTH-K2044. **(E)** *Galleria mellonella* larvae Kaplan–Meier survival analysis to assess the virulence *in vivo* of *K. pneumoniae* FK1979-WT or Φ-R mut2-7. Groups of 10 *G. mellonella* larvae were subjected to injection of 10^5^ CFU of *K. pneumoniae*. The blank control group was injected with 10 μL PBS. The survival rates of larvae infected with phage-resistant strains Φ-R mut2-7 were significantly higher compared with those injected with wild-type strain FK1979-WT and HvKP NUTH-K2044. **(F)** Kaplan–Meier survival curves for *K. pneumoniae* FK1979-WT or Φ-R mut2-7 infected mice. Mice were infected with 3 × 10^7^ CFU of different *K. pneumoniae* strains intraperitoneally. The previously reported hvKP strain NTUH-K2044, cKP strain ATCC700603 and saline were applied as the controls. Phage-resistant strains Φ-R mut2-7 showed lower virulence statistically significant from that of FK1979-WT and NTUH-K2044. No death of mice in the Φ-R mut2/3/5/7 or saline groups were observed during 7 days. **(G,H)** Biofilm formation ability of strains on a polystyrene surface at 24 h, measured by OD_570nm_ after crystal-violet staining (*n* = 6). The biofilm mass of phage-resistant strains Φ-R mut2-7 was significantly elevated than those of FK1979-WT and HvKP NUTH-K2044. All experiments were measured on three separate occasions. HvKP NUTH-K2044 as a positive control of virulence in all virulence phenotype assays. Statistical significance of the differences between the WT control group and the mutant groups was analyzed by repeated measures analysis of variance (ANOVA) **(A,B)**; Mann–Whitney test **(C,D)**; log-rank test **(E,F)**; and unpaired *t*-tests, two-tailed **(G)**. ^***^p < 0.001; n.s., no significance.

To gain insight into the fitness costs associated with phage resistance, the *in vitro* growth kinetics, serum resistance, adhesive to host cell, anti-phagocytosis by immune cell, *in vivo* infection in *G. mellonella* and mice infection models, and biofilm formation of resistant variants were compared with their wild-type. The mutant strains Φ-R mut2/3/5 exhibited dramatically slower growth dynamics compared with Φ-R mut4/6/7 and the parental strain FK1979-WT (Figure 7A). To gauge the influence of phage resistance-induced degradation of CPS on bacterial survival under serum exposure, complement bactericidal assays were performed. Mutants also correlated with the loss of serum resistance, while FK1979-WT and positive control of virulence hvKP NUTH-K2044 survived the exposure to human serum over a 3 h incubation period at 37°C (Figure 7B). To determine whether the loss of CPS production would affect bacterial invasiveness, the adhesion and phagocytosis assays were performed using RAW264.7 cells. The results revealed that the numbers of bacteria not washed out by PBS for mutants (~10^6^ CFU/well) were significantly higher than those of FK1979-WT and the virulence control strain NUTH-K2044 (10^4^ CFU/well; *p* < 0.001) and the numbers of intracellular bacteria for mutants (~10^5^ CFU/well) were approximately 2-log higher than those of the FK1979-WT and control strain NUTH-K2044 (10^3^ CFU/well; *p* < 0.001; Figure 7C). Similarly, the adhesion and internalization assays in the A549 cell also showed dramatically elevated numbers of bacteria in the cell or not washed out by PBS of Φ-R mut2-7 than the wild-type strain, indicating that phage-resistant mutants were more prone to adhesion and internalization by A549 cells compared to the FK1979-WT and control strain NUTH-K2044 (*p* < 0.001; Figure 7D). This result indicates that phage resistance can impair the capacity of hvKP to resist phagocytosis by host cells. Furthermore, *G. mellonella* larvae survival analysis was to assess the virulence *in vivo* of phage-resistant mutants. The result showed that the survival rates of *G. mellonella* within 72 h post-infection by Φ-R mut2/5/7 dramatically increased than FK1979-WT, indicating that phage resistance diminishes the virulence *in vivo* of hvKP (*p* < 0.001; Figure 7E). Similarly, the mice infection model showed that the mice inoculated with FK1979-WT or positive control NUTH-K2044 died within 1 or 2 days while no death of those with phage-resistant mutants including Φ-R mut2/3/5/7 was observed. The survival rates of mice infected with Φ-R mut4/6 or negative control strain ATCC700603 were also significantly higher than that with FK1979-WT. These results indicated that the *in vivo* virulence of phage-resistant mutants was dramatically decreased compared with FK1979-WT (*p* < 0.0001; Figure 7F). In addition, the biofilm biomass of phage-resistant mutants dramatically increased vs. the FK1979-WT (*p* < 0.001; Figures 7G,H). All in all, these results suggested that phage resistance attributed to a deficiency of CPS renders fitness costs of hvKP and thus sensitizes bacteria to serum complement, intracellular killing by immune cells and epithelial cells, as well as immune defense systems *in vivo*.

### 3.8. Characteristics of new phages targeting ΦFK1979-resistant mutants

**Figure 8:**
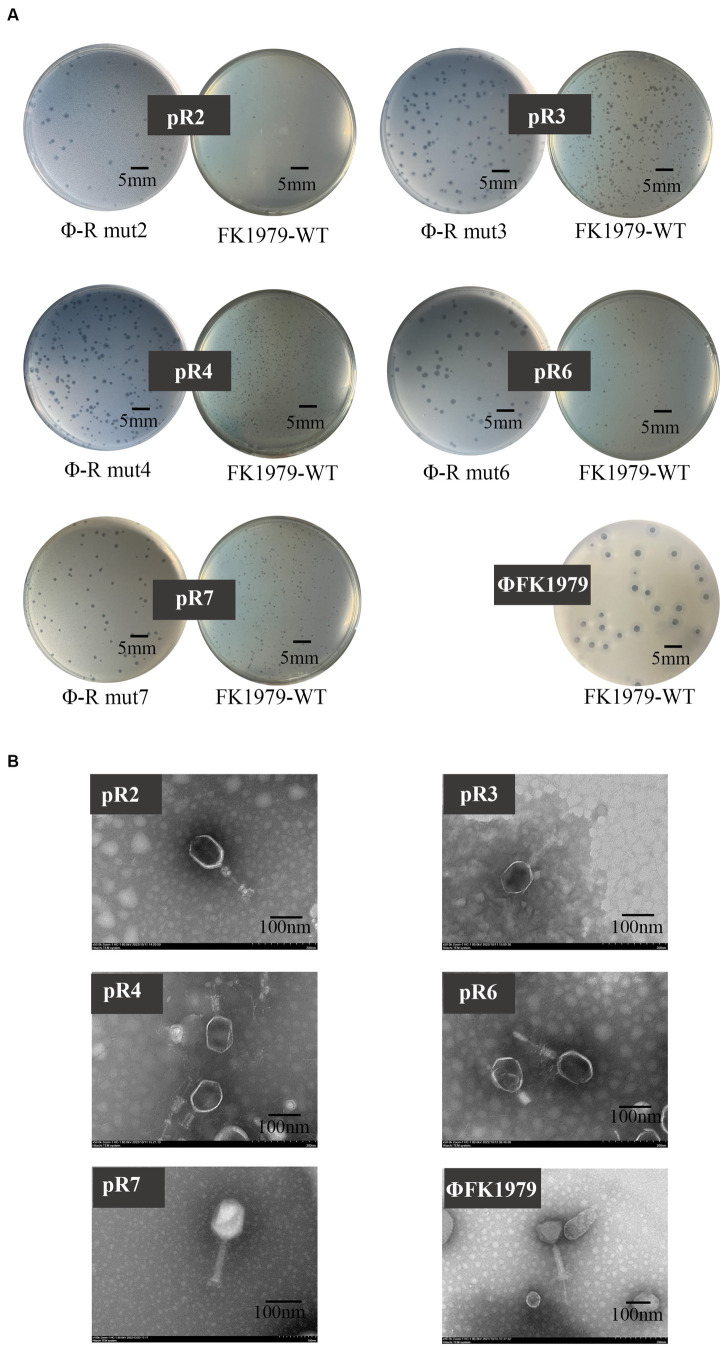
Plaque and TEM morphology of phages targeting *Klebsiella pneumoniae* FK1979-WT and ΦFK1979-resistant mutants isolated from hospital sewage. **(A)** Plaques of phages on their bacterial lawn. **(B)** TEM of phages.

We successfully isolated, from the same sewage, phages targeting Φ-R mut2/3/4/6/7 except for Φ-R mut5. Interestingly, plaque morphology showed that there were no typical turbid rings of the plaques of these newly isolated phages on either their host strains Φ-R mut2/3/4/6/7 or the wild-type strain FK1979-WT, significantly different from that of ΦFK1979 on the wild-type strain FK1979-WT (Figure 8A), which indicated the loss of CPS in ΦFK1979-resistant mutants. Further, the phage particle morphology under TEM showed that these newly isolated phages were somewhat different from ΦFK1979 in terms of their head and tail (Figure 8B). These new phages targeting Φ-R mut2/3/4/6/7, named pR2/pR3/pR4/pR6/pR7, were sequenced. Their relative information were listed in Table 3. Of note, the genomes of these phages were much larger than that of ΦFK1979. A larger genome generally means more complex and more proficient functions of phage. Genome annotation and bioinformatic analysis showed that phage pR2/pR3/pR4/pR6/pR7 possessed many more functional proteins, including structural proteins, replication and transcription proteins, and metabolic proteins. For example, phage pR2/pR3/pR4/pR6/pR7 encoded abundant baseplate proteins. In a bacteriophage, the baseplate is a specialized structure found at the bottom of the phage tail that plays a crucial role in the attachment of the phage to the host bacterial cell and the injection of the phage genetic material into the host cell. Furthermore, phage pR2/pR3/pR4/pR6/pR7 had more enzymes for replication and transcription, such as DNA polymerases, helicases, and primases, which ensure the faithful replication and expression of the viral genome and the lysis of bacteria to produce more phage particles. In addition, phage pR2/pR3/pR4/pR6/pR7 encoded more metabolic enzymes, including amino acid biosynthesis, nucleotide metabolism, sugar utilization and nutrient transporters. They endow the phage to rely on the metabolic machinery of the host bacteria to reproduce its genetic material and produce viral proteins. These properties of pR2/pR3/pR4/pR6/pR7 possibly help them to recognize receptors and lyse bacteria more efficiently (Supplementary Figure S3). The results of host spectrum tests of pR2/pR3/pR4/pR6/pR7 on different K-type clinical *K. pneumoniae* ascertained this hypothesis. As shown in Table 2, the phage pR2/pR3/pR4/pR6/pR7 could lyse 12 out of 15 K-type strains, except for K1, K5, K63 type, of *K. pneumoniae*. What is more interesting, pR3 seemed to have a significantly broader host spectrum than the other phages.

## 4. Discussion

In this study, we recovered a lysis phage, ΦFK1979, of the Drulisvirus genus able to rapidly and efficiently lyse *K. pneumoniae* FK1979. We demonstrated by both phenotype and genotype studies that FK1979 is a typical K2 ST86 hvKP highly producing CPS and ΦFK1979 encodes polysaccharide depolymerase specific to K2 capsule type *K. pneumoniae*, which indicated that ΦFK1979 is a promising agent for the treatment of invasive K2 hvKP infections. However, FK1979 rapidly developed resistance to ΦFK1979 during their co-incubation. The loss of effective absorption and lysis, and the same even after multiple passages indicated the formation and stability of phage-resistant mutants. This phenomenon represents a disadvantage for a therapeutic phage (Sumrall et al., 2019; Hesse et al., 2020; Tan D. et al., 2020). Fortunately, however, we found that there still was optimization space in its therapy for hvKP infection, through the revelation of the underlying mechanisms.

On the one hand, we revealed that phage resistance is gained at the cost of virulence and fitness in hvKP hosts. Attributed to the high production of CPS, the wild-type hvKP exhibits hypermucoviscous phenotype with the mucoid colony and positive string testing. However, in mutants, colony morphology changed from mucoid to rough, and the expression level of genes responsible for the biosynthesis or regulation of CPS and/or LPS dramatically decreased, indicating that CPS deficiency mediated phage resistance. Furthermore, the mutations in genes encoding rfaH [a conserved core contributor to CPS and LPS biosynthesis of *K. pneumoniae* (Nagy et al., 2002, 2006; Bachman et al., 2015; Dorman et al., 2018)], sugar glycosyltransferase and polysaccharide deacetylase, as well as galU, which are all essential in the regulation and biosynthesis of CPS and/or LPS, also unraveled the same phage resistance mechanisms of CPS decline in mutants. More and more studies have illustrated that the mutations in genes involved in the CPS and/or LPS synthesis contribute to phage resistance due to the loss of absorption to receptors (Burmeister et al., 2020; Tan D. et al., 2020; Gordillo Altamirano et al., 2021). All in all, the disruption of genes involved in bacterial surface polysaccharides resulted in the down-regulation or even absence of CPS and therefore rendering phage resistance. CPS has been demonstrated to be a key virulence factor for *K. pneumoniae* to escape antimicrobial agents or immune attacks (Stummeyer et al., 2006). The hydrophilicity and negative charge of CPS commonly limit complement deposition on the bacterial surface thus resistant to complement-mediated killing (Sharma et al., 2020). CPS plays an important role in resistance to phagocytosis and adhesion (Tan Y. H. et al., 2020). The CPS has been proven to be negatively associated with bacterial biofilm formation due to its effect on blocking the exposure of bacterial surface adhesins (Joseph and Wright, 2004; Junges et al., 2017). The diminished *in vitro* growth, serum resistance, and *in vivo* infection, as well as the strengthened biofilm formation and adhesion to or phagocytosis by the host cell, in phage-resistant mutants, can all be explained by its loss of the CPS. Several recent research has observed the same phenomenon (Mangalea and Duerkop, 2020; Tan D. et al., 2020; Kaszowska et al., 2021; Song et al., 2021). As such, although the absence or reduction of CPS rendered phage resistance, it simultaneously diminished virulence and fitness. The ascapsule low-virulent mutants can be killed by host immune systems *without the use of traditional antimicrobial therapy.* We proposed it to be the main benefit of phage ΦFK1979 therapy for hvKP infections*, as it disarms targeted pathogens and avoids the development of antimicrobial resistance* (Shen and Loessner, 2021). These results support that K2-specific phage can be used as an antivirulence agent to fight against hvKP.

On the other hand, newly isolated phages targeting resistant bacteria had significantly more functional proteins and much wider host spectrums than phage ΦFK1979. The bacteria have a dynamic self-regulation ability in the face of phages that recognize different receptors. Specifically, for capsule-targeting phages, CPS serves as the first receptor for its adsorption and cleavage; but for membrane protein-targeting phages, the existence of a capsule layer masks the membrane protein, causing resistance to it; Under phage resistance induced by capsule-targeting phages, capsule loss or synthesis deficiency favor phages targeting membrane proteins. Previous studies have also reached a similar conclusion (Majkowska-Skrobek et al., 2021). In the actual hvKP infection process, although the capsular-targeting phage may rapidly lyse sensitive bacteria, phage resistance is ubiquitous and seems inevitable, and the formation of phage-resistant bacteria in the late course of the disease may lead to unhealed or even aggravated infection. Based on this, single-or multi-phage therapy approaches, or different timing and order of phage administration may differ in the therapeutic effect. If capsule-targeting phages and phages-targeting resistant bacteria are used for phased administration of hvKP-infected individuals, the efficacy may be significantly improved. Especially for immunocompromised or deficient patients, although the resistant bacteria are more sensitive to the host’s immunity as we implied above, the compromised host immune cannot effectively kill the residual resistant bacteria. In this case, the addition of phages targeting resistant bacteria in the late course of the disease may yield considerable results. What is more, since host bacteria rarely develop resistance based on mutation of multiple targets at the same time, the combination of ΦFK1979 and phages targeting resistant bacteria may efficiently kill both the phage-sensitive and-resistant bacteria. In a study utilizing this to optimize phage combinations, the phage pairs consisting of one non-capsule targeting phage and one recognizing CPS have been found able to delay the emergence and growth of resistant clones in the *A. baumannii* population (Regeimbal et al., 2016). Bogna J. Smug et al. developed network prediction tools based on phage resistance to intelligently design phage cocktails against *K. pneumoniae* (Smug et al., 2022). Isolation and characterization of phages targeting resistant bacteria will facilitate the design of a phage cocktail for hvKP infections.

In conclusion, our study revealed that the absence or reduction of CPS and/or LPS rendered phage resistance in K2 ST86 hvKP under the pressure of K2 capsule-specific phage. However, the newly isolated phages targeting ΦFK1979-resistant mutants possessed much more functional proteins and much wider host spectrums. More importantly, the resistant bacteria were found associated with attenuation of virulence and fitness, thus more prone to be cleared by the immune system. These results suggest K2-specific phage as one part of phage cocktails or antivirulence agents to fight against hvKP, both of which are novel antimicrobial strategies of great clinical significance.

## Data availability statement

The datasets presented in this study can be found in online repositories. The names of the repository/repositories and accession number(s) can be found at: https://www.ncbi.nlm.nih.gov/, ON146449; https://www.ncbi.nlm.nih.gov/, CP094940; https://www.ncbi.nlm.nih.gov/, CP094941; https://www.ncbi.nlm.nih.gov/, SRR23217087; https://www.ncbi.nlm.nih.gov/, SRR23217088; https://www.ncbi.nlm.nih.gov/, SRR23217089; https://www.ncbi.nlm.nih.gov/, SRR23217090; https://www.ncbi.nlm.nih.gov/, SRR23217091; https://www.ncbi.nlm.nih.gov/, SRR23217092; https://www.ncbi.nlm.nih.gov/, OP684128; https://www.ncbi.nlm.nih.gov/, OR138016; https://www.ncbi.nlm.nih.gov/, OR138018; https://www.ncbi.nlm.nih.gov/, OR138017; and https://www.ncbi.nlm.nih.gov/, OR138015.

## Ethics statement

The animal study was reviewed and approved by the Laboratory Animal Ethics Committee of the First Affiliated Hospital of Wenzhou Medical University (WYYY-AEC-2022-047).

## Author contributions

MT: writing-original draft preparation, conceptualization, methodology, software, and visualization. ZH and XZ: conceptualization, methodology, software, formal analysis, and data curation. JK: investigation, formal analysis, and data curation. BZ: software, investigation, and validation. YH: methodology, software, and investigation. YZ: methodology and software. LC: resources, supervision, and project administration. TZ: writing-reviewing and editing, supervision, project administration, and funding acquisition. All authors contributed to the article and approved the submitted version.

## Funding

This work was supported by research grants from the National Natural Science Foundation of China (no. 82172328) and Key Laboratory of Clinical Laboratory Diagnosis and Translational Research of Zhejiang Province (2022E10022).

## Conflict of interest

The authors declare that the research was conducted in the absence of any commercial or financial relationships that could be construed as a potential conflict of interest.

## Publisher’s note

All claims expressed in this article are solely those of the authors and do not necessarily represent those of their affiliated organizations, or those of the publisher, the editors and the reviewers. Any product that may be evaluated in this article, or claim that may be made by its manufacturer, is not guaranteed or endorsed by the publisher.

## Footnotes

^1^ https://github.com/tseemann/snippy
