# Supplementary material for: Phage resistance formation and fitness costs of hypervirulent Klebsiella pneumoniae mediated by K2 capsule-specific phage and the corresponding mechanisms
Source: Front Microbiol. 2023 Jul 19;14:1156292. doi: 10.3389/fmicb.2023.1156292 (PMC10394836; doi:10.3389/fmicb.2023.1156292)
Supplement: Supplementary file 1 [file Data_Sheet_1.PDF]

Supplementary Material

1 Supplementary tables

1.1 **Table S1** Primers used in this study.

Primers	Sequence (5'-3')	Usage
galU-uF	TAGGGATAACAGGGTAATaatcttccgggccagttccc	Flanking up region for *galU* disruption
galU-uR	GAAGCAGCTCCAGCCTACACAttcgacgagtgcgttaccag	
galU-dF	CTAAGGAGGATATTCATATGctacatgcaggcgtttgtcg	Flanking down region for *galU* disruption
galU-dR	TAGGGATAACAGGGTAATcgcggcagaaattgaagagc	
galU-koconf_F	GCGGGATTAGGCACCAGGAT	To verify *galU* disruption
galU-koconf_R	CAGCATATCAATGGCGTCAGTCA	
rfaH-uF	TAGGGATAACAGGGTAATttgcttgaactgcggcagct	Flanking up region for *rfaH* disruption
rfaH-uR	GAAGCAGCTCCAGCCTACACAttccagatgttcctgggctc	
rfaH-dF	CTAAGGAGGATATTCATATGgatcttcaccgagcccgacg	Flanking down region for *rfaH* disruption
rfaH-dR	TAGGGATAACAGGGTAATaatttttccacgccgcacga	
rfaH-koconf_F	GCAAGCCTGGTACTTACTGTATT	To verify *rfaH* disruption
rfaH-koconf_R	CGGTGATTAGCACGCTATCG	
KanF	TGTGTAGGCTGGAGCTGCTTC	Amplified kan gene fragment from pKD4
KanR	CATATGAATATCCTCCTTAG	
qPCR-galU-F	TCCGATCTGAGCCGCGATAACC	Primers for RT-qPCR
qPCR- galU-R	TGCAGTCAACCACGCCATAAGC	
qPCR-rfaH-F	AACTGCCTGATGCCGACGATTG	Primers for RT-

qPCR-rfaH-R	GCGCTGATGGTGGTGGTATGAA	qPCR
qPCR-wzm-F	CCGGTCCGTGATTCCGCTAAGT	Primers for RT-qPCR
qPCR-wzm-R	CAAGGACGGCGTCATGCCATAA	
qPCR-wzt-F	AGTTGGCAAAGGTGAGGCTGTT	Primers for RT-qPCR
qPCR-wzt-R	GTCCTTCAGTGGTGACGGTTCC	
qPCR-wcaG-F	TGCCTACGCAGACGCTAACCT	Primers for RT-qPCR
qPCR-wcaG-R	CGGAATCTTCGGTGGAGAACGG	
qPCR-waaE-F	GCAGCACCGACAACACCGTT	Primers for RT-qPCR
qPCR-waaE-R	GAATGCCATAGCCTCGCCAGTC	
qPCR-wbbM-F	ATGCGGGTGAGAACAAACCA	Primers for RT-qPCR
qPCR-wbbM-R	AGCCGCTAACGACATCTGAC	
qPCR-pdfp-F	AGCGTGGATGAACAGCGTGAAG	Primers for RT-qPCR
qPCR-pdfp-R	CGTAGTCATTGCCCTGCGTCAA	
qPCR-sgtr-F	GCGTTGATTGAGGACGGTTGCT	Primers for RT-qPCR
qPCR-sgtr-R	CATCGCCGCATTCTGCTGGTAA	
qPCR-rcsA-F	AGGTCAACAGTGCTGCGGATCT	Primers for RT-qPCR
qPCR-rcsA-R	CAAACAGCGTCGCCGGGTTT	
qPCR-wzi-F	AACCAGCGCCTGTCTGCCTTA	Primers for RT-qPCR
qPCR-wzi-R	ACCACTCGCCGCTGTTGTTG	
qPCR-wcaJ-F	TTGTAGGACCTCGACCGCATG	Primers for RT-qPCR
qPCR-wcaJ-R	TCTGTTTCACCACGCCAACCA	

qPCR-galF-F	CAACCCGTTTGTGGTGGTCCTG	Primers for RT-qPCR
qPCR- galF-R	GGCGATCATAGCGGCGAGGTTA	
qPCR-rpoB-F	AAGGCGAATCCAGCTTGTTCAGC	Primers for RT-qPCR
qPCR-rpoB-R	TGACGTTGCATGTTCGCACCCATCA	
pACYC184-*galU*-F	CCACACCCGTCCTGTGGATCCgcaattgcgtttatatctaagcagacggga	Primers for construction of pACYC184-*galU*
pACYC184-*galU*-R	CGCCGCCGCAAGGAATGGTGCATGCttacttcgctaccgccgtttccag	

1.2 **Table S2** Strains and plasmids used in this study.

**Strain/plasmid**	**Characteristic**	**Source**
***K. pneumoniae* strains**		
FK1979	Wild type	This study
Φ-R mut5	Phage-resistant bacteria with *galU* mutant	This study
FK1979Δ*galU*	FK1979 with *galU* gene disrupted mutant	This study
FK1979Δ*rfaH*	FK1979 *rfaH*:: *km* deletion mutant	This study
Φ-R mut5*::galU*	*galU* complementary strain	This study
***Escherichia coli* strains**		
DH5α	Cloning strain	Takara
Plasmids		
pKD4	Source of FRT-flanked Km^r cassette; Amp^r Km^r	Chen L[a]
pACBSR	Mutagenesis plasmid used for gene gorging. Ara pro moter control, I-SceI and λ Red recombinase; Chl^r	Chen L[a]
pGEM-T Easy-*galU*:Km	pGEM-T Easy containing *galU* flanking sequences an d Km^r cassette used for gene gorging; Amp^r Km^r	This study
pGEM-T Easy-*rfaH*:Km	pGEM-T Easy containing *rfaH* flanking sequences an	This study

	d Km[r] cassette used for gene gorging; Amp[r] Km[r]	
pACYC184	Medium-copy-number, cloning vector, p15A ori; Tet[r] Chl[r]	Chen L[a]
pACYC184-*galU*	pACYC184 containing the FK1979 *galU* gene, Chl[r]	This study

[a] Chen L, Wilksch JJ, Liu H, Zhang X, Torres VVL, Bi W, Mandela E, Cao J, Li J, Lithgow T, Zhou T. Investigation of LuxS-mediated quorum sensing in Klebsiella pneumoniae. J Med Microbiol. 2020 Mar;69(3):402-413. doi: 10.1099/jmm.0.001148. PMID: 32223838; PMCID: PMC7377169.

1.3 **Table S3** Functional genes of phage ΦFK1979.

ORF no.	Function	Nucleotide ID	Sequence length (aa)	Accession number
1	Fiber_protein	312 2045	577	UPW35138.1
2	Endolysin	2056 2610	199	UPW35139.1
3	Holin	2639 2890	83	UPW35140.1
4	Spanin	2883 3287	134	UPW35141.1
5	Tail_protein	3287 3469	59	UPW35142.1
6	Fiber_protein/tail fiber protein	3481 3855	124	UPW35143.1
7	DNA maturase/terminase large subunit	3855 5711	618	UPW35144.1
9	tail fiber protein	6025 6948	307	UPW35146.1
10	internal core protein	6950 10669	1239	UPW35147.1
12	internal virion protein	13417 14004	195	UPW35149.1
13	tail tubular protein	14006 16387	793	UPW35150.1
14	tail tubular protein	16397 16957	186	UPW35151.1
15	Tail_protein/tail tip protein	17044 17376	110	UPW35152.1
16	transcriptional regulator	17426 17608	60	UPW35153.1
17	Head_protein/capsid protein	17620 18639	339	UPW35154.1
18	scaffolding protein	18665 19507	280	UPW35155.1
19	Head_protein/head-tail connector protein	19522 21117	531	UPW35156.1
23	DNA-dependent RNA polymerase	22199 24667	822	UPW35160.1
26	DNA endonuclease VII	25685 25897	70	UPW35164.1
29	5'-3' exonuclease	26485 27453	322	UPW35166.1
38	DNA polymerase	31224 33623	799	UPW35176.1
40	DNA helicase	34246 35526	426	UPW35179.1
41	DNA primase/helicase	35627 36436	269	UPW35181.1
44	peptidase	37097 38143	348	UPW35184.1
Others	Hypothetical_protein			

2 Supplementary Figures

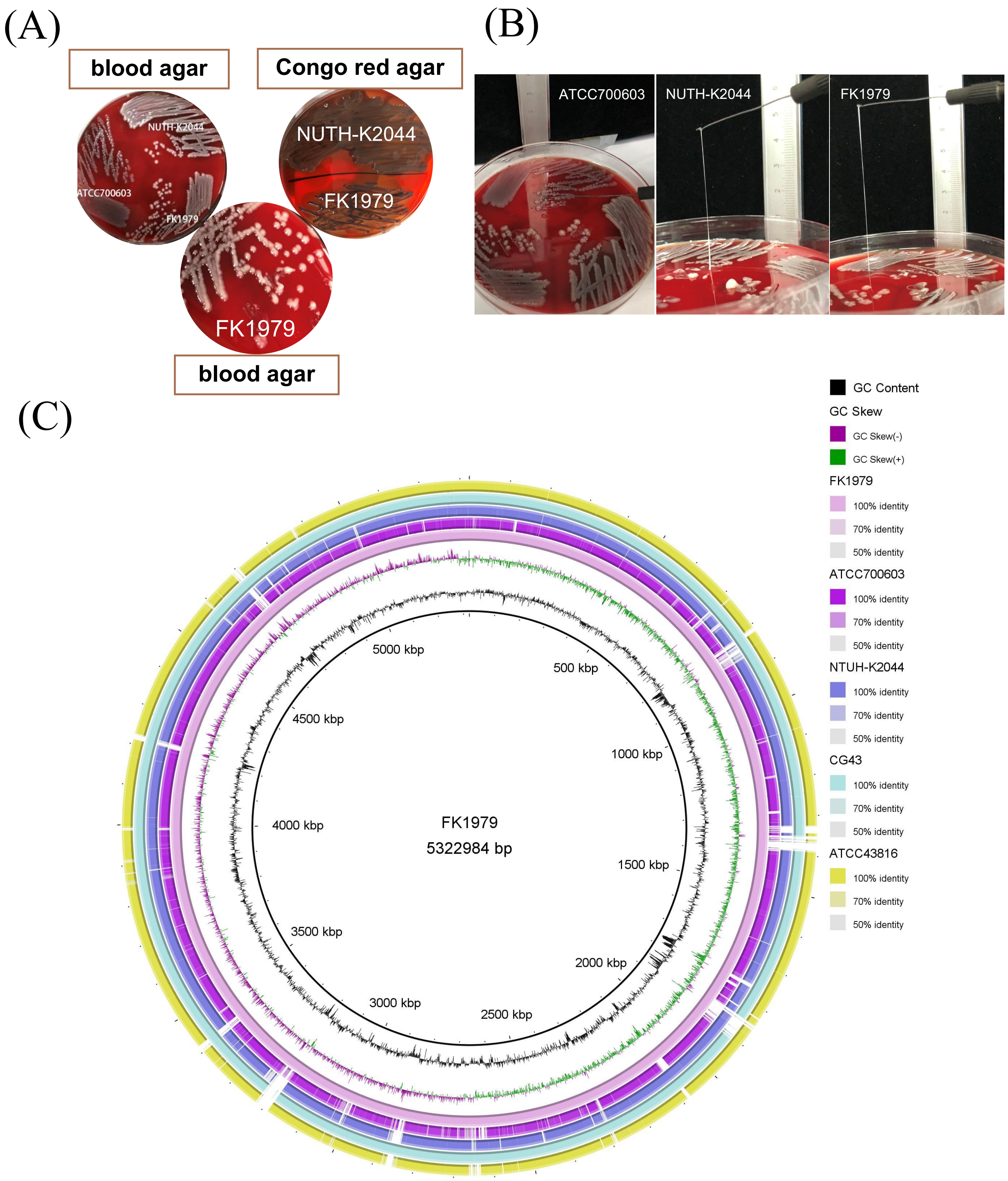

(A)
blood agar
Congo red agar
NUTH-K2044
ATCC700603
FK1979
NUTH-K2044
FK1979
FK1979
blood agar
(B)
ATCC700603
NUTH-K2044
FK1979
(C)
GC Content
GC Skew
GC Skew(-)
GC Skew(+)
FK1979
100% identity
70% identity
50% identity
ATCC700603
100% identity
70% identity
50% identity
NTUH-K2044
100% identity
70% identity
50% identity
CG43
100% identity
70% identity
50% identity
ATCC43816
100% identity
70% identity
50% identity
FK1979
5322984 bp
5000 kbp
500 kbp
4500 kbp
1000 kbp
4000 kbp
1500 kbp
3500 kbp
2000 kbp
3000 kbp
2500 kbp

(D)

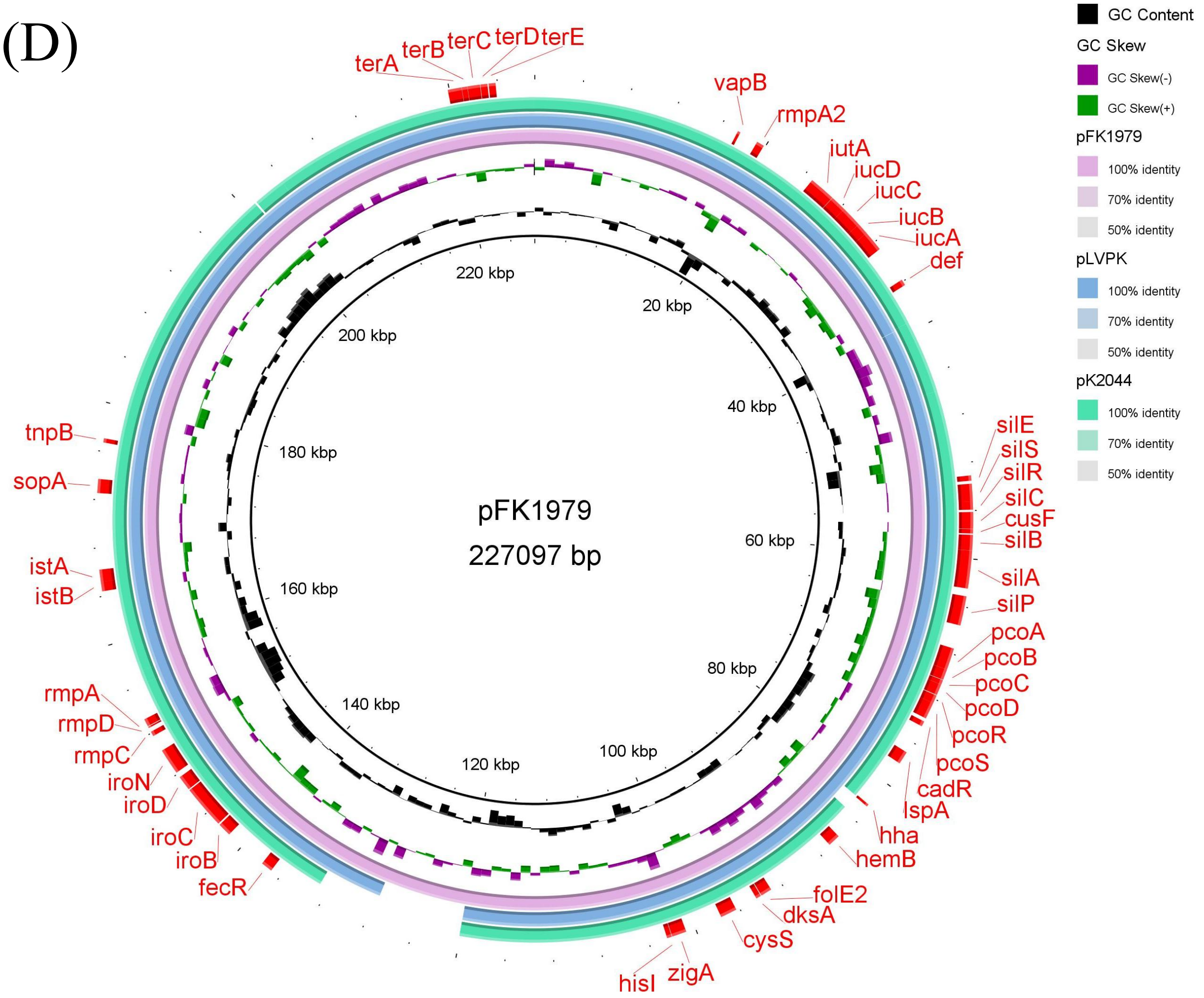

Figure S1 Phenotype and genomic characteristics of host *K. pneumoniae* FK1979. (A) Colony features compared with representative hypervirulent *K. pneumoniae* (hvKP) NUTH-K2044 and classic *K. pneumoniae* ATCC700603 on blood agar and Congo red agar. (B) String assay detecting hypermucoviscosity phenotype of FK1979. *K. pneumoniae* ATCC700603 and NUTH-K2044 as negative and positive control strains, respectively. (C) Comparative genome circle map of whole genomes of FK1979 with documented hvKP CG43, NUTH-K2044, ATCC43816 and classic *K. pneumoniae* ATCC700603. (D) Comparative plasmid genome circle map of pFK1979 with representative hypervirulent plasmid pK2044 (NC_006625.1) and pLVPK (NC_005249.1).

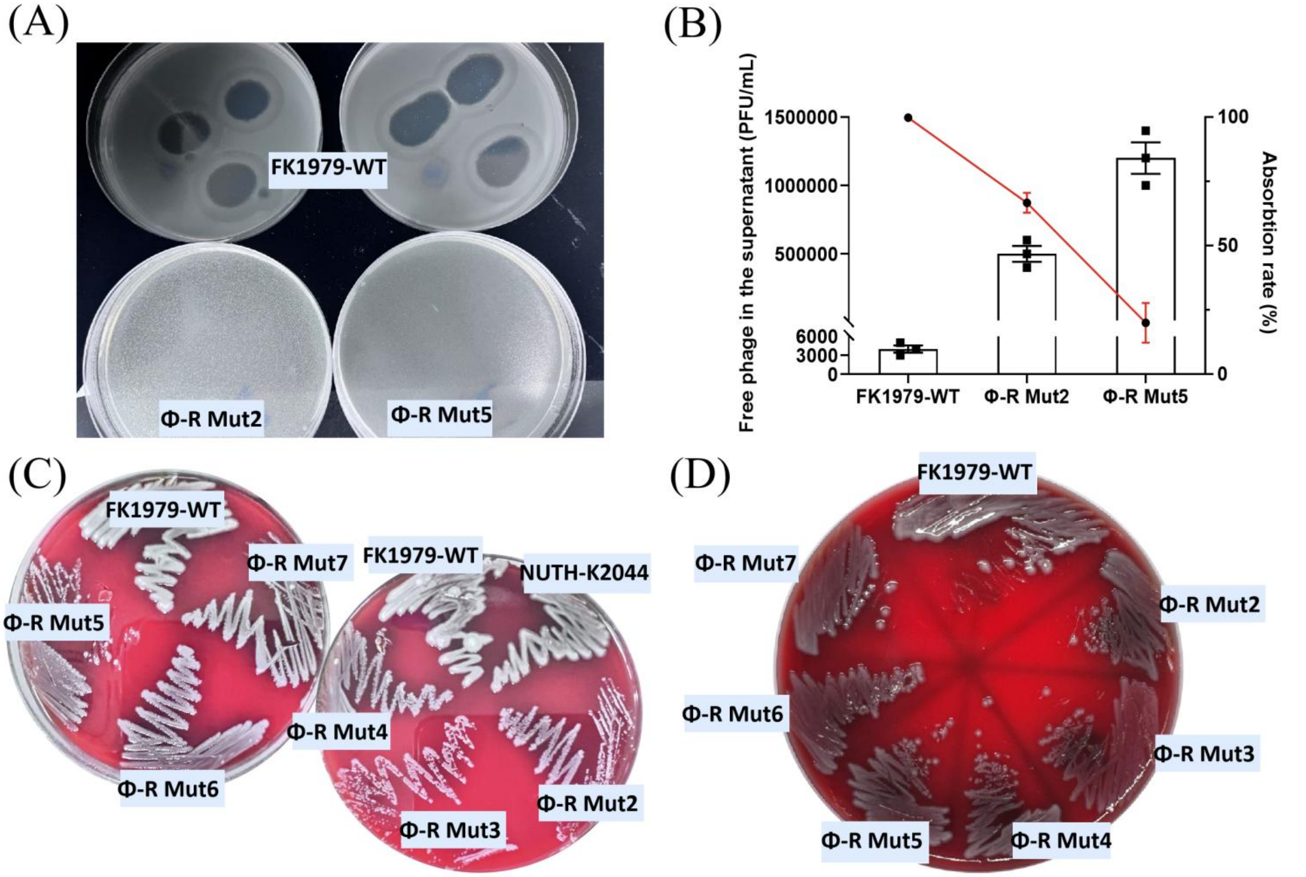

Figure S2 Confirmation of the phage-resistant mutant formation. (A) The spot test assays of phage ΦFK1979 against wild-type strain and phage-resistant mutants. (B) Adsorption assay of phage ΦFK1979 onto the FK1979-WT and phage-resistant strains. The bar chart reflects the titer of the free phage in the supernatants (left Y axis), and the line chart represents the adsorption rate of the phage ΦFK1979 by the strains (right Y axis). The results of phage-resistant mutants Φ-R Mut2 and Φ-R Mut5 as representatives were shown. Mann-Whitney test was used to assess the statistical significance between FK1979-WT and Φ-R Mut2/5. (C) Morphology comparison between colonies of *K. pneumoniae* on a blood agar plate. FK1979-WT and positive control hypervirulent *K. pneumoniae* (hvKP) NUTH-K2044 (mucoid, moist, and sticky) while phage-resistant mutants Φ-R mut2-7 (dry, rough, and transparent) at the first time of isolation. (D) Colony morphology after ten serial passages of the phage-resistant mutants (dry, rough, and transparent). FK1979-WT and hvKP NUTH-K2044 (mucoid, moist, and sticky) as control.

(A)

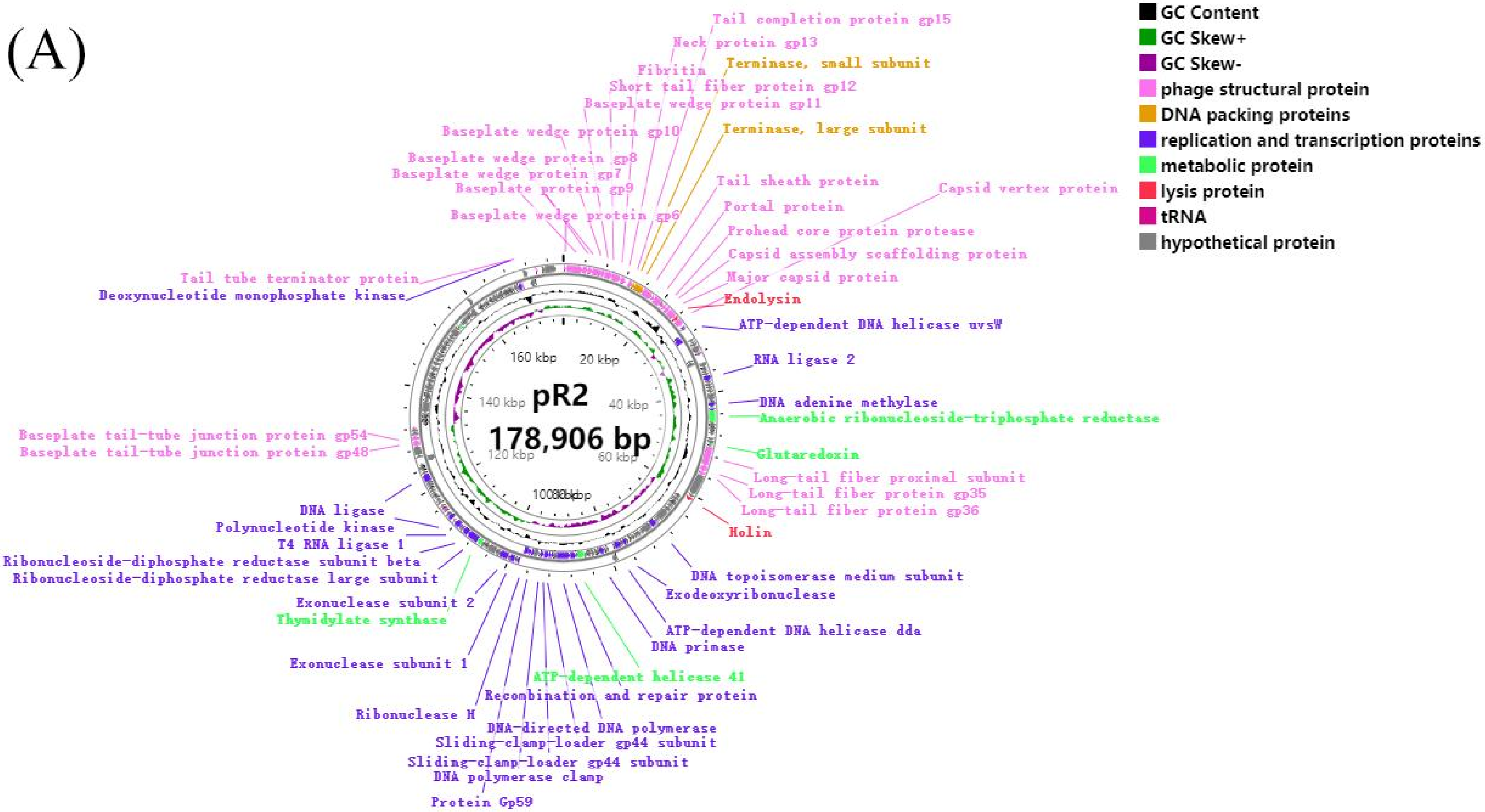

GC Content
GC Skew+
GC Skew-
phage structural protein
DNA packing proteins
replication and transcription proteins
metabolic protein
lysis protein
tRNA
hypothetical protein
pR2
178,906 bp
20 kbp
40 kbp
60 kbp
80 kbp
100 kbp
120 kbp
140 kbp
160 kbp
Tail completion protein gp15
Neck protein gp13
Fibritin
Terminase, small subunit
Short tail fiber protein gp12
Baseplate wedge protein gp11
Baseplate wedge protein gp10
Terminase, large subunit
Baseplate wedge protein gp8
Baseplate wedge protein gp7
Baseplate protein gp9
Baseplate wedge protein gp6
Tail sheath protein
Portal protein
Capsid vertex protein
Prohead core protein protease
Capsid assembly scaffolding protein
Major capsid protein
Endolysin
ATP-dependent DNA helicase uvsW
RNA ligase 2
DNA adenine methylase
Anaerobic ribonucleoside-triphosphate reductase
Glutaredoxin
Long-tail fiber proximal subunit
Long-tail fiber protein gp35
Long-tail fiber protein gp36
Holin
DNA topoisomerase medium subunit
Exodeoxyribonuclease
ATP-dependent DNA helicase dda
DNA primase
ATP-dependent helicase 41
Recombination and repair protein
DNA-directed DNA polymerase
Sliding-clamp-loader gp44 subunit
Sliding-clamp-loader gp44 subunit
DNA polymerase clamp
Protein Gp59
Ribonuclease H
Exonuclease subunit 1
Exonuclease subunit 2
Thymidylate synthase
Ribonucleoside-diphosphate reductase subunit beta
Ribonucleoside-diphosphate reductase large subunit
T4 RNA ligase 1
Polynucleotide kinase
DNA ligase
Baseplate tail-tube junction protein gp54
Baseplate tail-tube junction protein gp48
Tail tube terminator protein
Deoxynucleotide monophosphate kinase

(B)

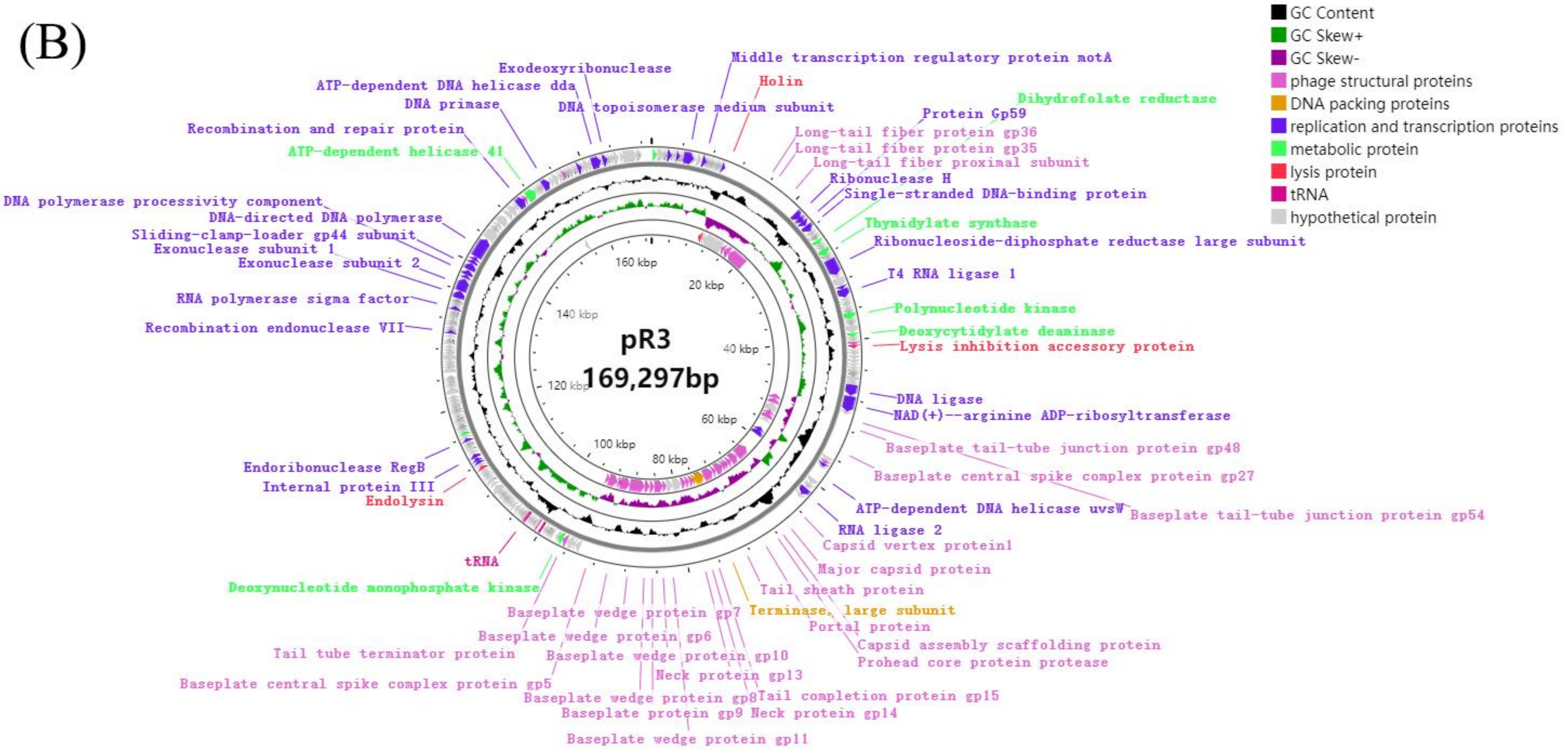
GC Content
GC Skew+
GC Skew-
phage structural proteins
DNA packing proteins
replication and transcription proteins
metabolic protein
lysis protein
tRNA
hypothetical protein
pR3
169,297bp
Middle transcription regulatory protein motA
Holin
Exodeoxyribonuclease
ATP-dependent DNA helicase dda
DNA primase
DNA topoisomerase medium subunit
Recombination and repair protein
ATP-dependent helicase 41
Dihydrofolate reductase
Protein Gp59
Long-tail fiber protein gp36
Long-tail fiber protein gp35
Long-tail fiber proximal subunit
Ribonuclease H
Single-stranded DNA-binding protein
DNA polymerase processivity component
DNA-directed DNA polymerase
Sliding-clamp-loader gp44 subunit
Exonuclease subunit 1
Exonuclease subunit 2
Thymidylate synthase
Ribonucleoside-diphosphate reductase large subunit
T4 RNA ligase 1
RNA polymerase sigma factor
Polynucleotide kinase
Recombination endonuclease VII
Deoxycytidylate deaminase
Lysis inhibition accessory protein
DNA ligase
NAD(+)--arginine ADP-ribosyltransferase
Baseplate tail-tube junction protein gp48
Endoribonuclease RegB
Internal protein III
Endolysin
Baseplate central spike complex protein gp27
ATP-dependent DNA helicase uvsW
Baseplate tail-tube junction protein gp54
RNA ligase 2
Capsid vertex protein1
tRNA
Major capsid protein
Deoxynucleotide monophosphate kinase
Tail sheath protein
Baseplate wedge protein gp7
Terminase, large subunit
Portal protein
Baseplate wedge protein gp6
Capsid assembly scaffolding protein
Tail tube terminator protein
Baseplate wedge protein gp10
Prohead core protein protease
Neck protein gp13
Baseplate central spike complex protein gp5
Baseplate wedge protein gp8
Tail completion protein gp15
Baseplate protein gp9
Neck protein gp14
Baseplate wedge protein gp11

(C)

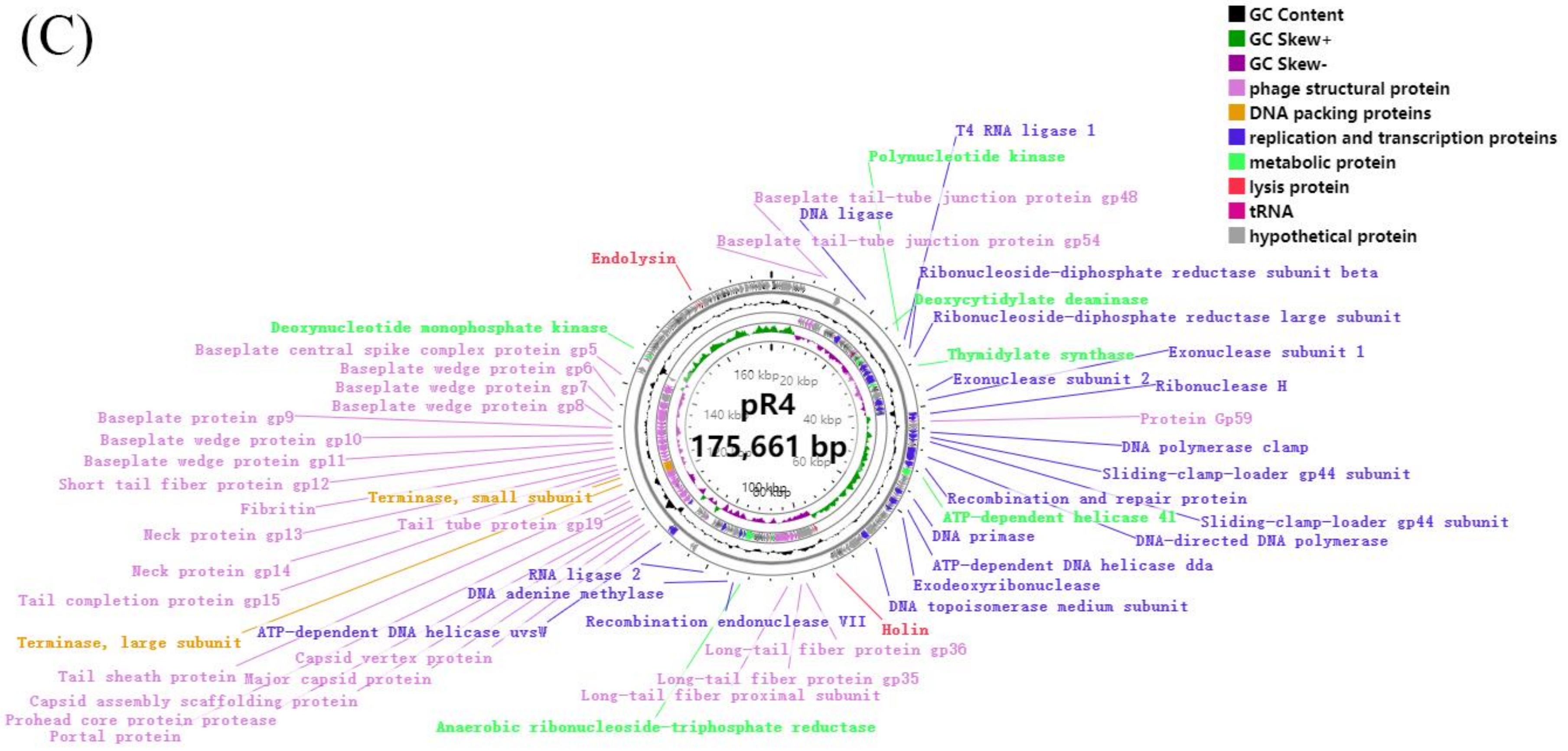
GC Content
GC Skew+
GC Skew-
phage structural protein
DNA packing proteins
replication and transcription proteins
metabolic protein
lysis protein
tRNA
hypothetical protein
pR4
175,661 bp
20 kbp
40 kbp
60 kbp
80 kbp
100 kbp
140 kbp
160 kbp
T4 RNA ligase 1
Polynucleotide kinase
Baseplate tail-tube junction protein gp48
DNA ligase
Baseplate tail-tube junction protein gp54
Endolysin
Ribonucleoside-diphosphate reductase subunit beta
Deoxycytidylate deaminase
Ribonucleoside-diphosphate reductase large subunit
Deoxynucleotide monophosphate kinase
Baseplate central spike complex protein gp5
Baseplate wedge protein gp6
Baseplate wedge protein gp7
Baseplate wedge protein gp8
Thymidylate synthase
Exonuclease subunit 1
Exonuclease subunit 2
Ribonuclease H
Baseplate protein gp9
Protein Gp59
Baseplate wedge protein gp10
DNA polymerase clamp
Baseplate wedge protein gp11
Sliding-clamp-loader gp44 subunit
Short tail fiber protein gp12
Terminase, small subunit
Recombination and repair protein
Fibritin
ATP-dependent helicase 41
Sliding-clamp-loader gp44 subunit
Tail tube protein gp19
DNA primase
DNA-directed DNA polymerase
Neck protein gp13
ATP-dependent DNA helicase dda
Neck protein gp14
RNA ligase 2
Exodeoxyribonuclease
DNA adenine methylase
Tail completion protein gp15
DNA topoisomerase medium subunit
Recombination endonuclease VII
Holin
ATP-dependent DNA helicase uvsW
Terminase, large subunit
Capsid vertex protein
Long-tail fiber protein gp36
Tail sheath protein
Major capsid protein
Long-tail fiber protein gp35
Capsid assembly scaffolding protein
Long-tail fiber proximal subunit
Prohead core protein protease
Portal protein
Anaerobic ribonucleoside-triphosphate reductase

(D)

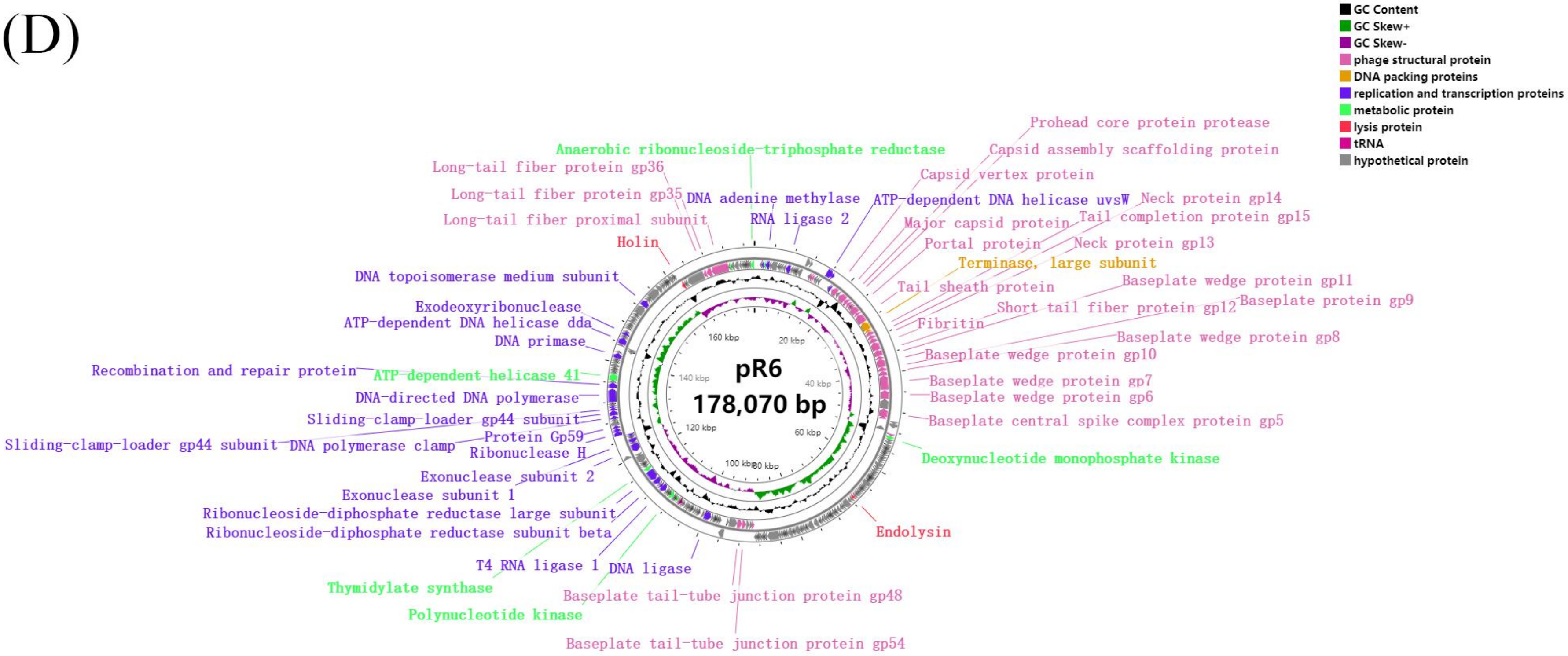
GC Content
GC Skew+
GC Skew-
phage structural protein
DNA packing proteins
replication and transcription proteins
metabolic protein
lysis protein
tRNA
hypothetical protein
pR6
178,070 bp
160 kbp
20 kbp
40 kbp
60 kbp
80 kbp
100 kbp
120 kbp
140 kbp
Prohead core protein protease
Capsid assembly scaffolding protein
Anaerobic ribonucleoside-triphosphate reductase
Long-tail fiber protein gp36
Capsid vertex protein
Long-tail fiber protein gp35
DNA adenine methylase
ATP-dependent DNA helicase uvsW
Neck protein gp14
Long-tail fiber proximal subunit
RNA ligase 2
Major capsid protein
Tail completion protein gp15
Holin
Portal protein
Neck protein gp13
Terminase, large subunit
DNA topoisomerase medium subunit
Tail sheath protein
Baseplate wedge protein gp11
Exodeoxyribonuclease
Short tail fiber protein gp12
Baseplate protein gp9
ATP-dependent DNA helicase dda
Fibritin
DNA primase
Baseplate wedge protein gp8
Baseplate wedge protein gp10
Recombination and repair protein
ATP-dependent helicase 41
Baseplate wedge protein gp7
DNA-directed DNA polymerase
Baseplate wedge protein gp6
Sliding-clamp-loader gp44 subunit
Baseplate central spike complex protein gp5
Protein Gp59
Sliding-clamp-loader gp44 subunit
DNA polymerase clamp
Ribonuclease H
Deoxynucleotide monophosphate kinase
Exonuclease subunit 2
Exonuclease subunit 1
Ribonucleoside-diphosphate reductase large subunit
Ribonucleoside-diphosphate reductase subunit beta
Endolysin
T4 RNA ligase 1
DNA ligase
Thymidylate synthase
Baseplate tail-tube junction protein gp48
Polynucleotide kinase
Baseplate tail-tube junction protein gp54

(E)

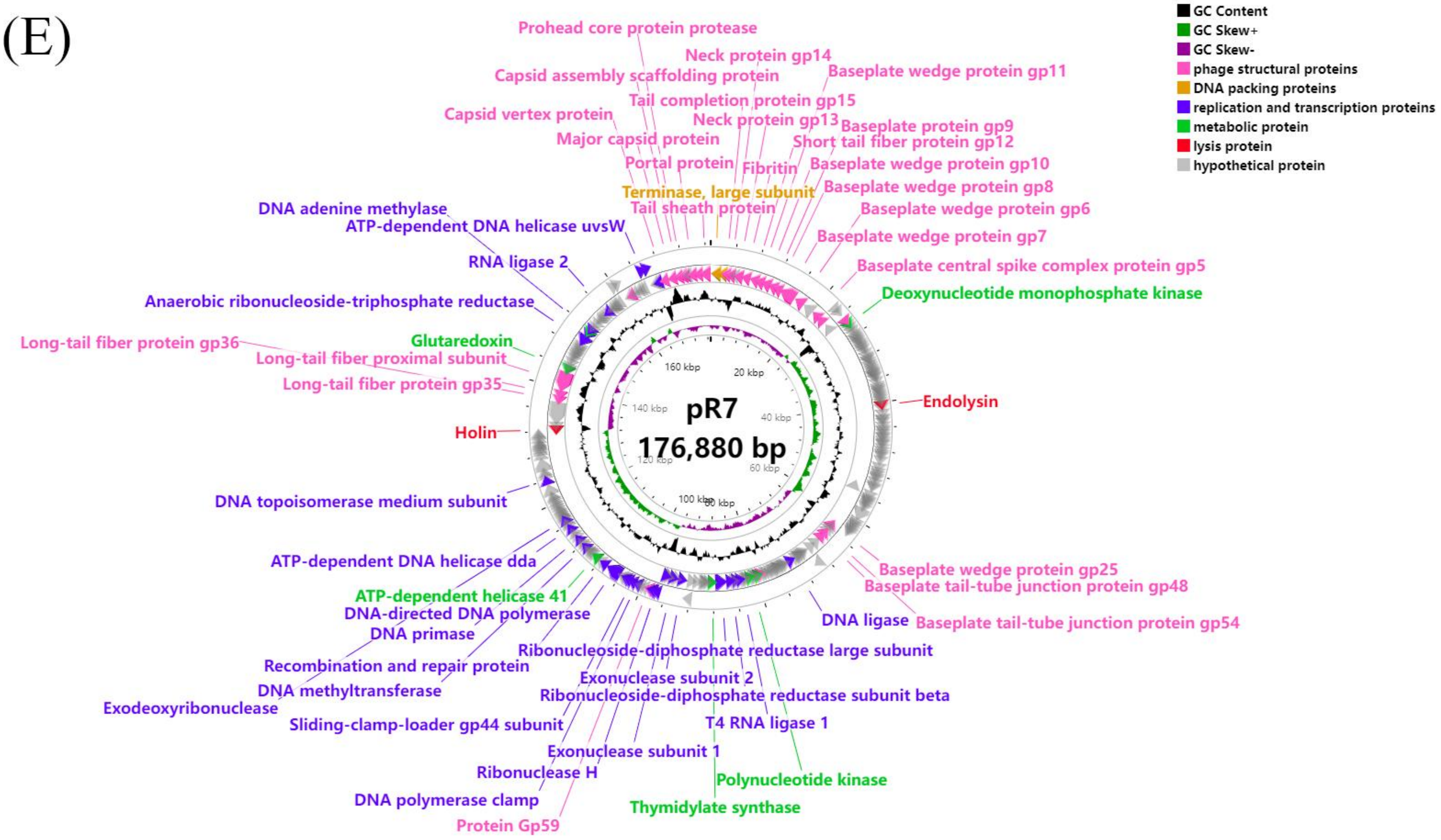
GC Content
GC Skew+
GC Skew-
phage structural proteins
DNA packing proteins
replication and transcription proteins
metabolic protein
lysis protein
hypothetical protein
pR7
176,880 bp
20 kbp
40 kbp
60 kbp
80 kbp
100 kbp
120 kbp
140 kbp
160 kbp
Prohead core protein protease
Neck protein gp14
Capsid assembly scaffolding protein
Baseplate wedge protein gp11
Tail completion protein gp15
Capsid vertex protein
Neck protein gp13
Baseplate protein gp9
Major capsid protein
Short tail fiber protein gp12
Portal protein
Fibritin
Baseplate wedge protein gp10
Terminase, large subunit
Baseplate wedge protein gp8
DNA adenine methylase
Tail sheath protein
Baseplate wedge protein gp6
ATP-dependent DNA helicase uvsW
Baseplate wedge protein gp7
RNA ligase 2
Baseplate central spike complex protein gp5
Deoxynucleotide monophosphate kinase
Anaerobic ribonucleoside-triphosphate reductase
Long-tail fiber protein gp36
Glutaredoxin
Long-tail fiber proximal subunit
Long-tail fiber protein gp35
Endolysin
Holin
DNA topoisomerase medium subunit
ATP-dependent DNA helicase dda
Baseplate wedge protein gp25
Baseplate tail-tube junction protein gp48
ATP-dependent helicase 41
DNA-directed DNA polymerase
DNA ligase
Baseplate tail-tube junction protein gp54
DNA primase
Ribonucleoside-diphosphate reductase large subunit
Recombination and repair protein
Exonuclease subunit 2
DNA methyltransferase
Ribonucleoside-diphosphate reductase subunit beta
Exodeoxyribonuclease
Sliding-clamp-loader gp44 subunit
T4 RNA ligase 1
Exonuclease subunit 1
Ribonuclease H
Polynucleotide kinase
DNA polymerase clamp
Thymidylate synthase
Protein Gp59

(F)

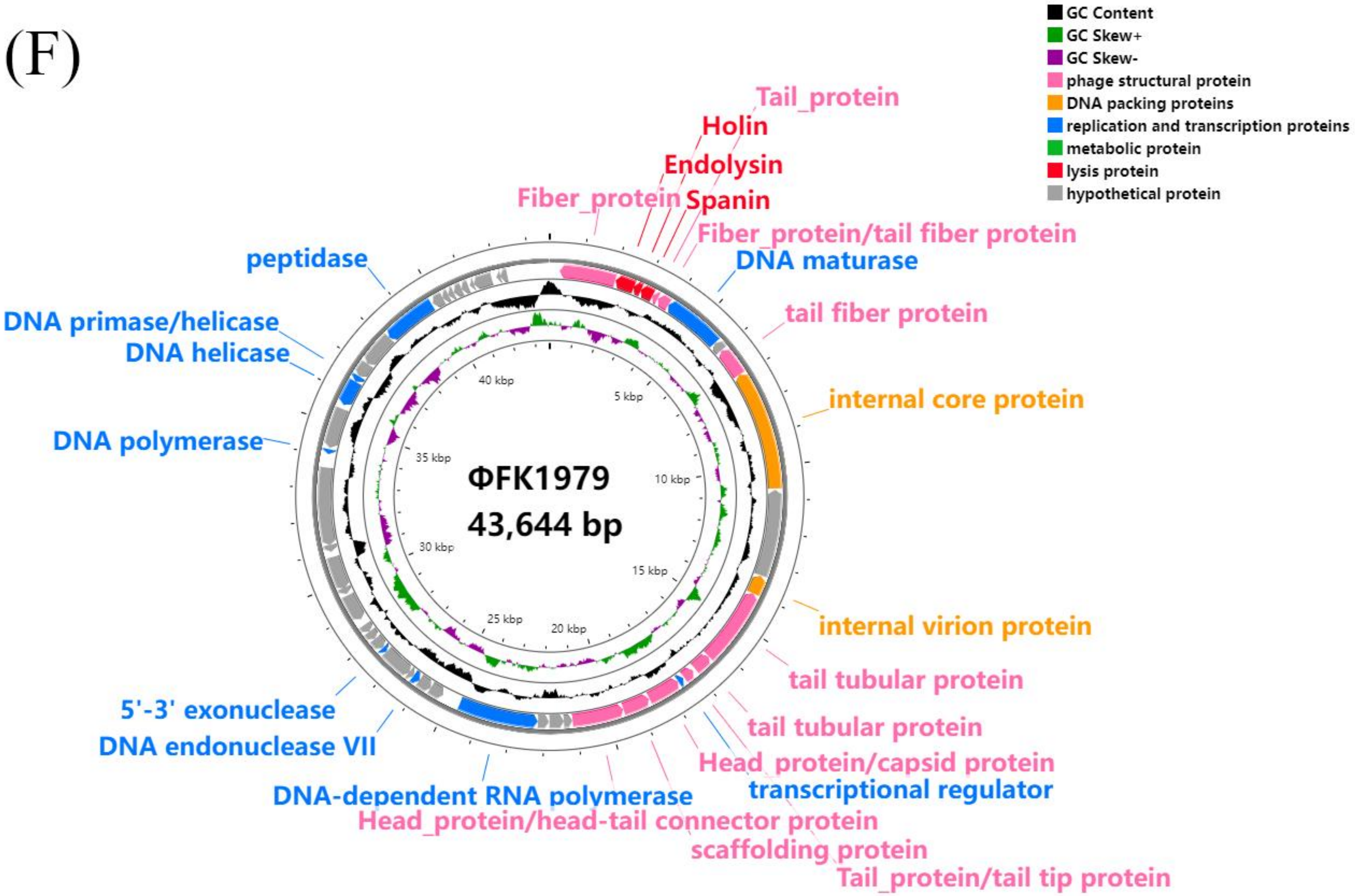

Figure S3 Genome map of the phage pR2 (A) pR3 (B) pR4 (C) pR6 (D) pR7 (E) compared with ΦFK1979 (F). The predicted ORFs and direction of transcription are indicated by arrows. Genes are colored based on function classification. Pink represents phage structural protein, blue represents phage replication and transcription proteins, DNA packing proteins are colored orange, phage metabolic proteins are colored green and lysis proteins are colored red. Genes are labeled with their products.
